# Cardiovascular Morbidity Following Definitive Chemoradiotherapy for Lung Cancer in an Appalachian Population

**DOI:** 10.3390/cancers18172830

**Published:** 2026-09-01

**Authors:** Vivian Salama, Joseph A. Schmidlen, John Christopher Knoth, Troy Nguyen, Aaron Naveen Joseph, Matthew Trotta, Ramon Alfredo Siochi, Raymond R. Raylman, Jeffrey Ryckman, Mohammed Almubarak, Christopher M. Bianco, David A. Clump, Mina F. Hanna, Phillip M. Pifer

**Affiliations:** 1 Department of Radiation Oncology, WVU Cancer Institute, West Virginia University School of Medicine, Morgantown, WV 26506, USA; joseph.schmidlen@hsc.wvu.edu (J.A.S.); john.knoth@hsc.wvu.edu (J.C.K.); rasiochi@hsc.wvu.edu (R.A.S.); jeff.ryckman@hsc.wvu.edu (J.R.); dclump@hsc.wvu.edu (D.A.C.); ppifer@hsc.wvu.edu (P.M.P.); 2 Department of Medical Oncology, WVU Cancer Institute, West Virginia University School of Medicine, Morgantown, WV 26506, USA; malmubarak@hsc.wvu.edu; 3 Department of Radiology, West Virginia University School of Medicine, Morgantown, WV 26506, USA; troy.nguyen@hsc.wvu.edu (T.N.); rraylman@hsc.wvu.edu (R.R.R.); mina.hanna@hsc.wvu.edu (M.F.H.); 4 Department of Internal Medicine, UPMC Medical Education, University of Pittsburgh Medical Center, Pittsburgh, PA 15213, USA; josephan4@upmc.edu; 5 Department of Radiation Oncology, The University of Alabama at Birmingham, Birmingham, AL 35233, USA; 6 Department of Cardiology and Cardiac Surgery, West Virginia University School of Medicine, Morgantown, WV 26506, USA; christopher.bianco@wvumedicine.org

**Keywords:** Appalachia, cardio-oncology, cardiovascular toxicity, chemoradiotherapy, lung cancer, mortality, morbidity

## Abstract

Background/Objectives: Cardiovascular morbidity following definitive chemoradiotherapy (CRT) for lung cancer is a major concern in Appalachia due to high rates of smoking and pre-existing cardiovascular disease. This study characterized the cardiovascular burden following CRT in a real-world Appalachian population. Methods: A retrospective study was conducted among Appalachian patients with lung cancer treated with definitive CRT from 2013 to 2025. Cardiovascular adverse event (CVAE) incidence, spectrum, and temporal distribution were characterized. Kaplan–Meier and Cox regression analyses evaluated time to CVAE and overall survival (OS). Exploratory machine-learning (ML) models assessed CVAE and mortality prediction. Results: Fifty-one patients (59%) experienced a CVAE. NSTEMI (15/51, 29%) and pericardial disease (15/51, 29%) were most frequent, followed by arrhythmia (8/51, 16%). Ischemic events occurred predominantly within the first year, whereas pericardial events were concentrated within 2 years. Median CVAE-free survival was 22.6 months, with estimated cumulative CVAE occurrence of 28.3% at 1 year and 50.0% at 2 years. After adjustment for age, cardiovascular comorbidity, and M stage, heart V20 (HR, 1.024 per 1% increase; 95% CI, 1.005–1.044; *p* = 0.015) and V30 (HR, 1.031; 95% CI, 1.004–1.058; *p* = 0.025) were associated with increased CVAE hazard. Median OS was 29 months; pre-existing cardiovascular comorbidity was associated with mortality (HR range, 2.16–2.45). Exploratory ML showed limited CVAE prediction but moderate mortality discrimination after feature selection (maximum AUC, 0.71). Conclusions: Cardiovascular morbidity was common and accumulated over time following definitive CRT in this Appalachian cohort. These findings support attention to baseline cardiovascular risk, cardiac radiation exposure, and longitudinal cardiovascular surveillance.

## Simple Summary

Patients with lung cancer often receive a combination of chemotherapy and radiation therapy, which improves survival but can also expose the heart to injury. That risk may be particularly high in Appalachian communities, where smoking, cardiovascular disease, and other health disparities are common. However, little is known about the frequency and types of heart problems experienced by these patients in routine clinical practice. In this study, we evaluated cardiovascular complications in Appalachian patients with lung cancer treated with definitive chemoradiotherapy and explored whether routinely collected clinical and radiation treatment information could help identify patients at increased risk. More than half of the patients developed at least one cardiovascular complication during or after treatment, highlighting the need for careful cardiovascular monitoring. These findings provide important real-world evidence that may inform treatment planning, cardiovascular surveillance, and future strategies to reduce heart complications in high-risk populations.

## 1. Introduction

Lung cancer remains the leading cause of cancer-related mortality in the United States [1]. Radiotherapy (RT) is commonly utilized in the curative-intent setting. Definitive chemoradiotherapy (CRT) serves as a cornerstone of treatment and has demonstrated improved overall survival (OS) in patients with locally advanced non-small-cell lung carcinoma (LA-NSCLC) [2] and limited-stage small-cell lung carcinoma (LS-SCLC) [3]. As survival improves, post-treatment cardiovascular morbidity has become increasingly important, particularly because lung cancer and cardiovascular disease (CVD) share risk factors such as tobacco smoking, hypertension, diabetes, and even socioeconomic factors [4,5]. Because of the close proximity of critical thoracic organs to the primary tumor, patients receiving CRT remain at risk for acute and late treatment-related toxicities, including esophageal, pulmonary, and cardiovascular toxicity (CVT) [6].

Cardiovascular toxicity (CVT) following CRT is a well-recognized and clinically important consequence of CRT in thoracic malignancies [7,8,9,10]. Toxicities following treatment can manifest acutely or months to years after exposure. CVT includes ischemic events, arrhythmia, congestive heart failure (CHF), and valvular disease, among others [11]. Studies investigating cardiac toxicity were primarily conducted in breast, esophageal, and Hodgkin lymphoma survivors [12,13,14]. Because of the proximity of the heart in thoracic tumors and the multimodality nature of treatment, patients with lung cancer are exposed to both cardiac irradiation and potentially cardiotoxic systemic therapy. Moreover, patients with lung cancer often carry a significant smoking history and pre-treatment cardiovascular comorbidity that has been associated with cardiovascular complications following treatment [15]. The multifactorial nature of cardiovascular adverse events (CVAEs) in lung cancer complicates treatment planning and personalized cardiovascular surveillance.

Patient factors and dosimetric relationships to CVT in patients receiving CRT have been investigated [15,16]; however, because the prevalence of baseline cardiovascular disease varies across studies, published estimates may underestimate risk in populations with a high pre-treatment burden. In a combined analysis of four prospective trials for LA-NSCLC, pre-existing cardiac disease was associated with a nearly three-fold increase in the risk of grade >3 cardiac events [15]. However, only a quarter of patients in this study had pre-existing cardiac disease, limiting applicability to populations with higher cardiovascular risk. Another study found that a higher radiation mean heart dose (MHD) correlated with worse OS; however, specific cardiac event endpoints were not investigated [17]. Both cohorts were primarily treated with 3D-conformal radiotherapy (3D-CRT), so their risk estimates may not apply to more modern techniques, such as intensity-modulated radiation therapy (IMRT).

These uncertainties are particularly relevant in Appalachia, a region with high rates of CVD [18,19], tobacco use [20], and lung cancer incidence [1,20]. Patients in this region often carry a substantial baseline cardiovascular risk profile, raising concerns that existing literature may underestimate the true burden of CVT in underserved populations. Traditional statistical approaches, which assume linear relationships and prespecified interactions, may not fully capture the complex interplay among clinical comorbidities, radiation dosimetry, and survival outcomes. Artificial intelligence and machine learning (AI/ML) approaches offer the potential to model nonlinear and multicollinear relationships and higher-order interactions, particularly for outcome prediction or feature importance identification following CRT [21,22,23]. However, studies exploring these novel technologies using real-world data in Appalachian populations are lacking.

Cardiovascular adverse events following CRT may arise from multiple contributing factors, including baseline CVD, smoking history, systemic therapy, and cardiac radiation exposure. To our knowledge, no prior study has characterized the cardiovascular burden following definitive CRT in an Appalachian lung cancer cohort. Characterizing the incidence, spectrum, and temporal occurrence of CVAEs in this population may improve recognition of cardiovascular morbidity during and after cancer treatment and inform multidisciplinary cardiovascular assessment and longitudinal surveillance in Appalachia and other medically underserved regions. Therefore, the primary objective of this study was to characterize the incidence, spectrum, and temporal burden of CVAEs following definitive CRT in a real-world Appalachian lung cancer cohort. Secondary objectives were to evaluate patient, clinical, and cardiac dosimetric factors associated with increased CVAEs and mortality risk in this population and to explore the potential predictive information provided by these routinely available characteristics using ML approaches. The ML analyses were considered exploratory and hypothesis-generating rather than intended to develop a clinically deployable prediction model. We hypothesized that this medically vulnerable Appalachian population would experience a substantial burden of cardiovascular morbidity following definitive CRT.

## 2. Materials and Methods

### 2.1. Study Design and Data Source

This institutional review board (IRB)-approved retrospective cohort study included consecutive adult patients with biopsy-confirmed lung cancer treated with definitive CRT at our institution between 2013 and 2025.

Eligible patients were identified by querying the institutional radiation oncology electronic medical record and planning systems including ARIA and Eclipse (Varian Medical Systems, Palo Alto, CA, USA). Demographic, clinical, and treatment-related data were extracted from the institutional electronic health record (Epic; Epic Systems Corporation, Verona, WI, USA) and the treatment planning systems.

### 2.2. Study Population

Patients were included if they: (1) were ≥18 years of age, (2) had biopsy-proven lung cancer, (3) were treated with definitive-intent CRT to the primary lung tumor and mediastinal nodal metastasis if present, (4) had complete treatment and dosimetric data available, (5) were treated using contemporary RT techniques, including three-dimensional conformal radiotherapy (3DCRT), intensity-modulated radiotherapy (IMRT), or volumetric modulated arc therapy (VMAT). Eligible disease categories included locally advanced non-small-cell lung cancer (LA-NSCLC), limited-stage small-cell lung cancer (LS-SCLC), and select oligometastatic NSCLC treated with definitive CRT.

Patients were excluded if they: (1) received non-definitive or palliative treatment, (2) had lung metastases from a non-pulmonary primary tumor, (3) had early-stage NSCLC treated with surgery, stereotactic body radiotherapy (SBRT), or hypofractionated RT, (4) received more than one prior course of thoracic RT before a documented CVAE (to minimize the potential influence of unaccounted cumulative radiation exposure). Patients were followed from the first day of RT until the first documented cardiovascular event, death, or last known clinical follow-up. The median follow-up was 30.5 months (IQR 13.8–47.0).

**Figure 1:**
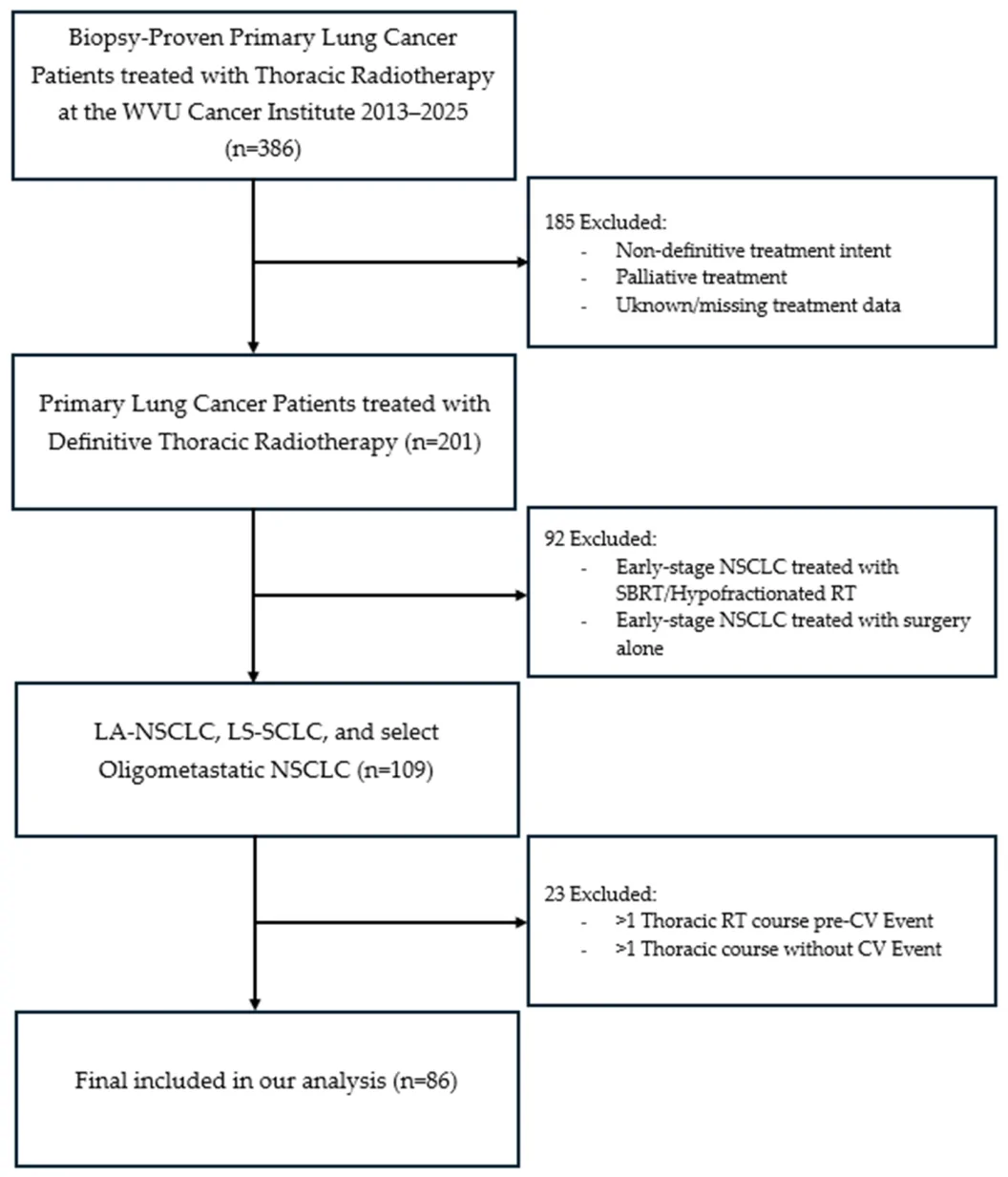
Flow chart of study population.

After applying eligibility criteria, 86 patients were included in the final analysis. A study flow diagram is presented in Figure 1. The study sample included all eligible patients treated during the study period and therefore no formal sample size calculation was performed.

### 2.3. Features/Variables

**Figure 2:**
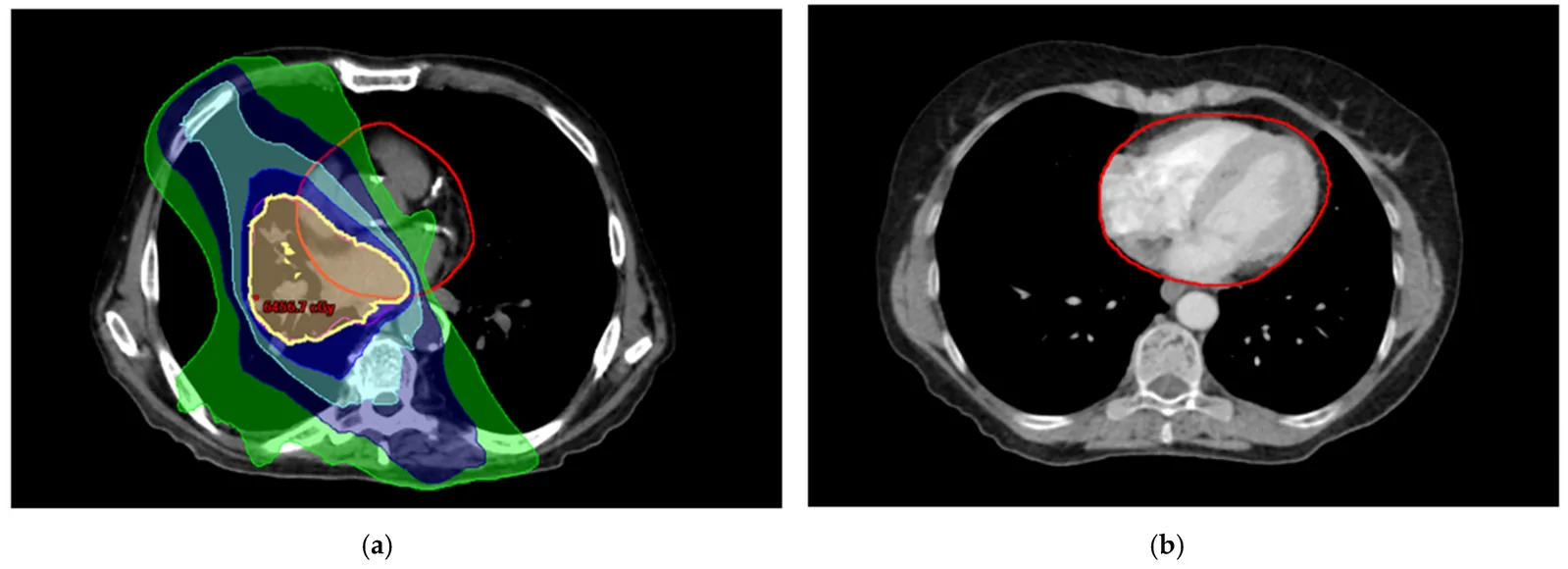
External beam radiation treatment plan in lung cancer. (**a**) Non-contrast CT simulation planning of an Appalachian patient diagnosed with lung cancer (NSCLC) showing the 60 (yellow), 50 (dark blue), 40 (light blue), 30 (purple), 20 (green) Gy Isodose Lines (IDLs) w/PTV (pink), and whole-heart contour (red). (**b**) Contrast CT image of another patient showing whole-heart contours (red).

Candidate features were prespecified based on clinical relevance and published literature. Variables included: age at diagnosis, sex, and race/ethnicity; smoking status (current, former, never); pre-existing cardiovascular comorbidity (defined as any documented coronary artery disease (CAD), heart failure, hypertension, cerebrovascular accident, peripheral artery disease, or arrhythmia before receiving RT); primary tumor site; histology and staging; treatment variables (e.g., surgery, chemotherapy, immunotherapy, total RT dose and number of fractions); and cardiac dosimetric parameters extracted from the treatment planning system [mean heart dose (MHD), heart V20(%), heart V30(%), heart V40 (%), and heart V50 (%)]. The whole heart was contoured on planning CT simulation images, and whole-heart dosimetric parameters were extracted from the Eclipse treatment planning system. Figure 2 illustrates a finalized radiation treatment plan from our institution. All predictor variables were measured prior to or at the time of CRT planning.

### 2.4. Study Outcomes

The primary outcome in this study was the occurrence of any CVAE during or after radiation treatment, dichotomized as [0 = No-CVAE and 1 = Yes-CVAE]. The composite CVAE endpoint included clinically documented cardiovascular events comprising non-ST-elevation myocardial infarction (NSTEMI), ST-elevation myocardial infarction (STEMI), arrhythmia, congestive heart failure (CHF), pericardial disease, cardiac death, new cardiology referral for concerning cardiac symptoms (i.e., chest pain), valvular dysfunction, new clinical diagnosis of CAD, and other major structural or functional cardiac abnormalities (e.g., ventricular aneurysm or newly reduced ejection fraction). A cardiology referral was included only when initiated during or after CRT for newly developed cardiac symptoms or an objectively documented abnormality, such as a new electrocardiographic abnormality or imaging finding. Events were counted only when a formal diagnosis was documented in the EHR and supported by a physician note or imaging report. All CVAEs included in the primary endpoint represented newly developed cardiovascular events occurring during or following definitive CRT. Cardiovascular conditions documented before treatment were classified as baseline cardiovascular comorbidities and were not considered CVAEs. Exacerbations of pre-existing comorbidities were not counted as events (e.g., an acute exacerbation after treatment in a patient with CHF diagnosed before RT). The composite endpoint was used to characterize the overall burden of clinically documented cardiovascular morbidity occurring during and after CRT in this real-world cohort rather than to imply equivalent clinical severity or pathophysiology among individual CVAE types. Although these events have distinct pathophysiologic mechanisms, a broad CVAE endpoint was used to provide a comprehensive assessment of cardiovascular events during or following definitive CRT. This composite endpoint was intended to characterize the overall spectrum of post-treatment cardiovascular burden rather than imply equivalence among individual event types. Individual CVAEs were subsequently characterized by clinical phenotype and timing following treatment.

The secondary outcome was patient mortality, dichotomized as (0 = alive and 1 = deceased). Survival was determined using institutional EHRs and confirmed through documented date of death, when applicable. Survival time was calculated in months from the day of RT initiation to death, or last known follow-up.

### 2.5. Statistical Analysis

#### 2.5.1. Descriptive Statistics

Categorical variables were summarized as numbers (*n*) and percentages (%). Continuous variables were summarized as mean ± standard deviation (SD) or median with interquartile range (IQR), as appropriate. Differences between patients with and without CVAEs were compared using the Wilcoxon test for numeric variables and the Chi-square test or Fisher’s exact test for categorical variables, as appropriate.

#### 2.5.2. Time-to-CVAE Analysis

Time to first CVAE was calculated from initiation of RT to the first documented new CVAE and categorized into predefined intervals of 0–6 months, 6–12 months, 1–2 years, 2–5 years, and >5 years. Patients without a CVAE were censored at their last available follow-up or death. CVAE-free survival was estimated using the Kaplan–Meier method, with the median CVAE-free survival and event-free probabilities at 6, 12, 24, 36, and 60 months reported.

Time to first new CVAE was also evaluated using Cox proportional hazards regression. Univariable Cox regression was initially performed to identify factors associated with CVAE risk. Multivariable models included variables significant in univariable analysis together with clinically relevant covariates, including age at diagnosis and pre-existing cardiovascular comorbidity. Because of substantial correlation among cardiac dosimetric parameters (Supplementary Figure S1), MHD and heart V20, V30, V40, and V50 were evaluated separately in individual adjusted models. Hazard ratios (HRs) with 95% confidence intervals (CIs) were reported.

#### 2.5.3. Survival Analysis

Overall survival (OS) was evaluated using time-to-event methods and was calculated in months from RT initiation to death or last known follow-up. The Kaplan–Meier method was used to estimate the survival distribution, median OS, and survival probabilities at 12, 24, 36, and 60 months.

Cox proportional hazards regression was used to evaluate associations between baseline clinical, disease-related, treatment, and cardiac dosimetric variables and OS. Candidate variables were initially evaluated using univariable Cox regression. Variables that demonstrated an association with OS in univariable analysis, together with clinically relevant covariates, were considered for multivariable models given the number of observed deaths and the modest cohort size. Hazard ratios (HRs) with 95% confidence intervals (CIs) and two-sided *p*-values were reported.

Separate adjusted Cox models were constructed for each cardiac dosimetric parameter to reduce potential multicollinearity and evaluate its association with OS after adjustment for selected clinical and disease-related covariates. The proportional hazards assumption was assessed for the final Cox models.

A 2-sided *p*-value of less than 0.05 was considered statistically significant in all statistical analyses.

#### 2.5.4. Exploratory Machine Learning Analysis

Twenty candidate predictors were initially included in ML model training, followed by feature selection to evaluate model performance using a reduced set of the most important features and clinically relevant variables. Missingness was quantified for each variable. Missing numerical values were imputed using the median and standardized using z-score normalization, while missing categorical values were imputed using the most frequent category and ordinally encoded.

Four supervised ML classifiers were evaluated: Gradient Boosting Machine (GBM), Random Forest (RF), Logistic Regression (LR), and Support Vector Machine (SVM). Hyperparameters were optimized using grid search where appropriate, while model complexity was constrained to reduce overfitting given the small sample size. Model development and internal validation were performed using five-fold stratified cross-validation with shuffling (random seed = 42).

Model performance was evaluated using the area under the receiver operating characteristic curve (AUC). Ninety-five percent confidence intervals were estimated using bootstrap resamples of the out-of-fold predictions.

Permutation feature importance was used to assess predictor importance for the best-performing tree-based models. Feature importance was quantified as the mean decrease in AUC following 30 random permutations of each predictor. External validation was not performed because of the limited sample size and will be pursued in future multi-institutional studies.

Following initial model development, feature selection was performed using permutation feature importance together with clinical relevance to identify a reduced set of predictors, and the models were subsequently re-evaluated using these selected features.

Statistical analyses were performed using Python version 3.13.2 (scikit-learn version 1.8.0), JMP Student Edition version 18.2.1, and GraphPad Prism version 10.5.0.

## 3. Results

### 3.1. Patient and Therapeutic Characteristics

A total of 86 Appalachian patients with lung cancer treated with definitive CRT were included. The cohort included 46 females (53%) and 40 males (47%). The mean age at diagnosis was 66 years (SD = 9.13; 95% CI: 64.0–68.0), with a range of 39 to 87 years. The majority (82/86, 95%) were either former or current smokers. Out of the 86 patients, 64 (74%) had pre-treatment cardiovascular comorbidities, 57/86 (66.3%) had hypertension, 30/86 (34.9%) had coronary artery disease, 16/86 (18.6%) had conduction disorder/arrhythmia, and 7/86 (8.1%) had CHF. The median RT total dose was 60 Gy (range: 44–70 Gy; IQR: 60–60). The median number of fractions was 30 (range: 22–35; IQR: 30–30).

Carboplatin/paclitaxel was the most frequently administered concurrent chemotherapy regimen (*n* = 42, 49%); 13% (*n* = 11) received cisplatin/etoposide, 12% (*n* = 10) received carboplatin/etoposide, 9% (*n* = 8) received carboplatin/pemetrexed, 6% (*n* = 5) received carboplatin only, 2% (*n* = 2) received carboplatin/etoposide/pembrolizumab, 1% (*n* = 1) received carboplatin/pemetrexed/pembrolizumab, 2% (*n* = 2) did not receive systemic therapy, and 6% (*n* = 5) received multiagent systemic therapy. Throughout treatment, 77% (*n* = 66) received immunotherapy.

Table 1 provides a summary of the cohort characteristics and the results of the Chi-square and Wilcoxon tests.

Cardiac dose–volume parameters showed the following: mean heart dose (Gy): median = (11.8), range = 0.06–32.24, IQR = 5.70–14.88; heart V50 (%): median = 3.72, range = 0–33.33, IQR = 0.03–10.73; heart V40 (%): median = 3.72, range = 0–33.33, IQR = 1.03–10.73; heart V30 (%): median = 9.77, range = 0–50.07, IQR = 2.36–17.51; and heart V20 (%): median = 20.31, range = 0–67.24, IQR = 6.255–29.89. Detailed dosimetric analyses of the patients’ CVAE groups and mortality status are summarized in Supplementary Table S1.

### 3.2. Incidence and Spectrum of Cardiovascular Adverse Events

Overall, 51 patients (59%) developed a CVAE during or after RT, whereas 35 patients (41%) did not experience a CVAE. At the time of last follow-up, 34 patients (40%) were alive, and 52 patients (60%) were deceased.

**Figure 3:**
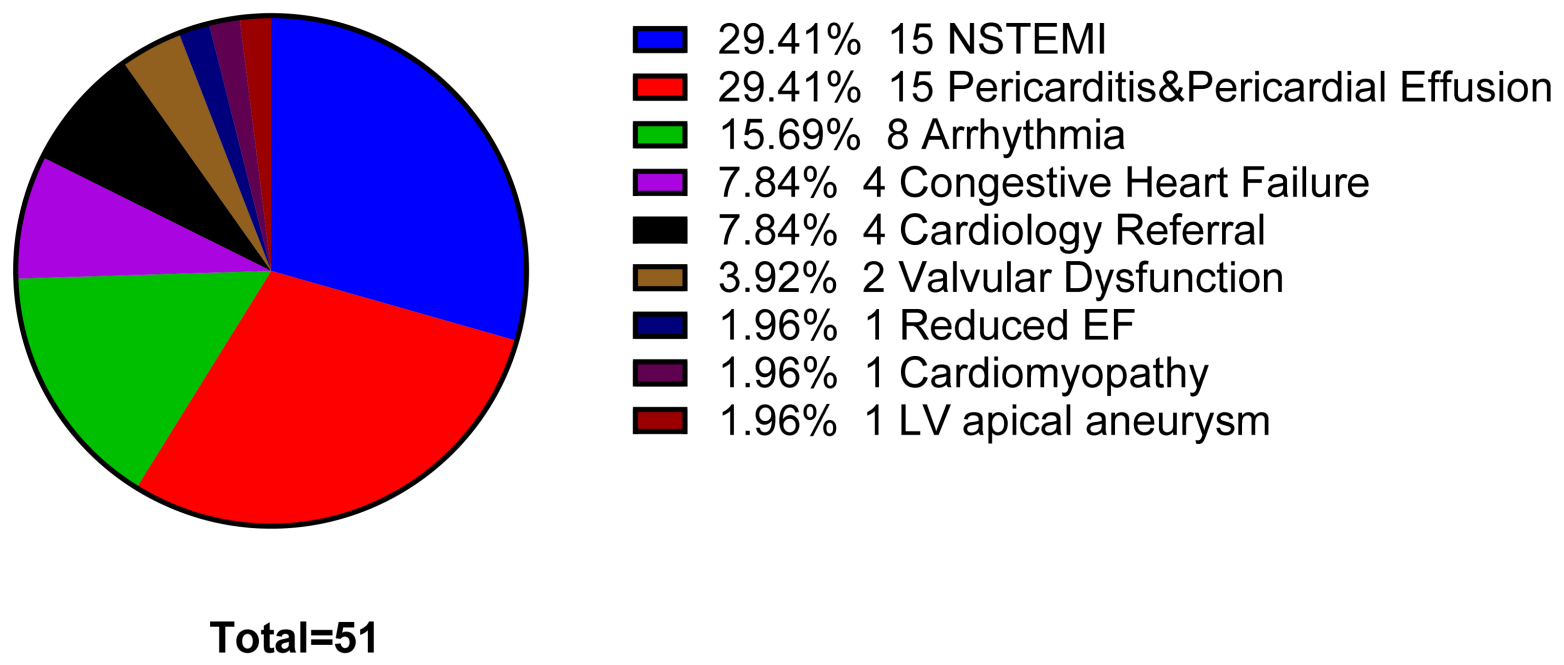
Frequency and types of cardiovascular adverse events during and after CRT in Appalachian patients with lung cancer.

Among the 51 patients who developed a CVAE during or after RT, the most common events were NSTEMI in 15 patients (29.41%) and pericarditis or pericardial effusion in 15 patients (29.41%), followed by arrhythmia in eight patients (15.69%). Less frequent events included CHF in four patients (7.84%), cardiology referral in four patients (7.84%), and valvular dysfunction in two patients (3.92%). Rare CVAEs included mild reduction in ejection fraction (one patient, 1.96%), cardiomyopathy (one patient, 1.96%), and left ventricular apical aneurysm (one patient, 1.96%) (Figure 3).

Temporal patterns of CVAEs differed by event category and type (Table 2 and Supplementary Table S2). Ischemic events (i.e., NSTEMI) occurred predominantly during the first year following RT initiation, with 60.0% occurring within 12 months. Pericardial-related events were concentrated within the first 2 years, with 33.3% occurring within 6 months and 46.7% at 1–2 years. Of the 15 pericardial events, two were clinically symptomatic pericarditis, whereas the remaining 13 were asymptomatic pericardial effusions incidentally detected on follow-up imaging. Arrhythmias showed a more distributed temporal pattern, with the largest proportion occurring at 2–5 years (37.5%). Events occurring beyond 5 years were uncommon and consisted primarily of NSTEMI and congestive heart failure.

### 3.3. Time-to-CVAE Analysis

**Figure 4:**
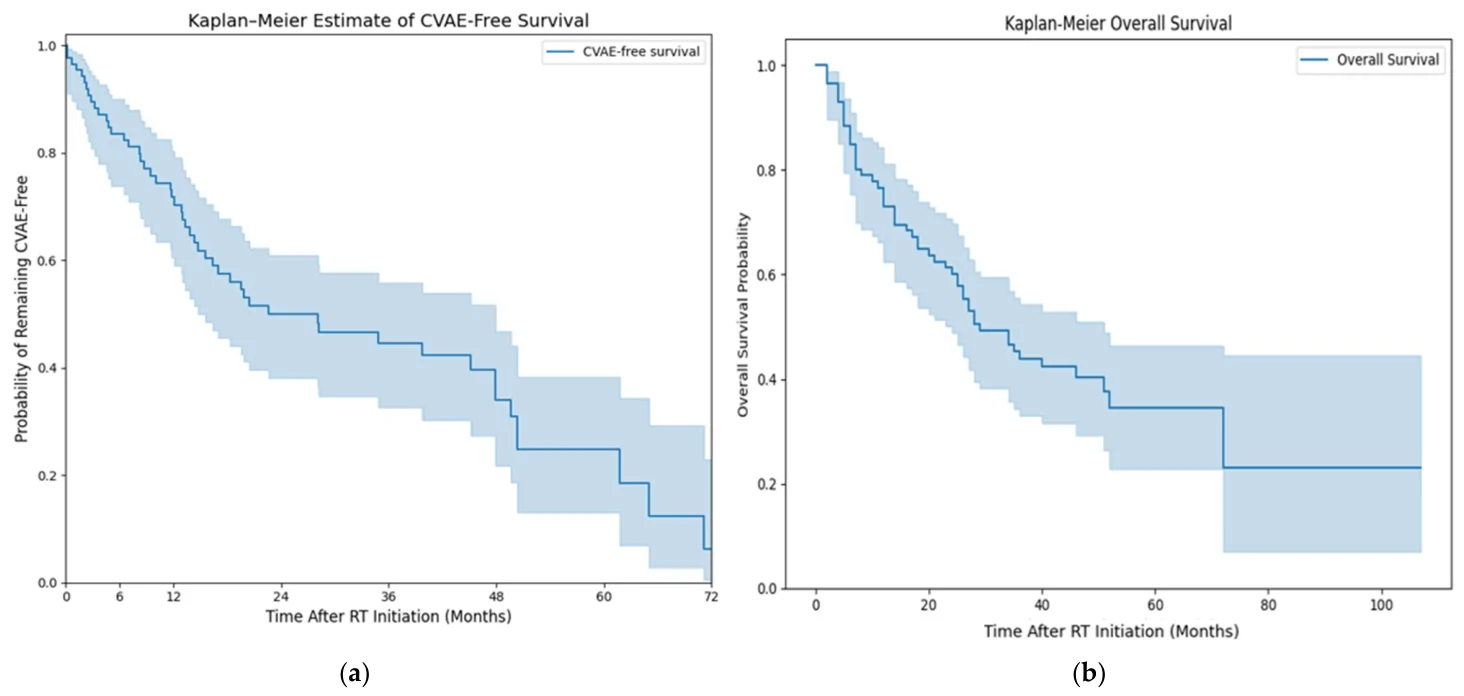
Kaplan–Meier curves of time to event. (**a**) Kaplan–Meier estimate of cardiovascular adverse event (CVAE) free survival. (**b**) Kaplan–Meier curve of overall survival. Solid blue lines represent Kaplan–Meier survival estimates, and shaded blue areas represent 95% confidence intervals.

The median CVAE-free survival was 687 days (22.6 months). Estimated CVAE-free survival rates at 6, 12, 24, 36, and 60 months were 83.5%, 71.7%, 50.0%, 44.5%, and 24.7%, respectively. Corresponding cumulative CVAE occurrence estimates were 16.5%, 28.3%, 50.0%, 55.5%, and 75.3% (Figure 4a).

Among the 51 patients who developed a CVAE, the median time to first CVAE was 396 days (13.0 months) (IQR, 149.5–771 days) after RT initiation. The mean time to CVAE was 585.7 days (19.2 months) ± 574.2 days (18.9 months), with events occurring from 5 to 2166 days (5.93 years) after treatment.

In univariable Cox regression, M stage was associated with an increased hazard of CVAE (HR, 4.50; 95% CI, 1.05–19.37; *p* = 0.043). The site of primary (HR, 1.75; 95% CI, 0.99–3.12; *p* = 0.056) and heart V20 (HR, 1.017 per 1% increase; 95% CI, 0.998–1.036; *p* = 0.073) demonstrated an increase in CVAE hazard (Supplementary Table S3).

In multivariable Cox models adjusted for age at diagnosis, cardiovascular comorbidity, and M-stage category, cardiac dosimetric parameters were evaluated separately. Higher heart V20 was independently associated with increased CVAE hazard (HR, 1.024 per 1% increase; 95% CI, 1.005–1.044; *p* = 0.015). Heart V30 was similarly associated with CVAE hazard (HR, 1.031 per 1% increase; 95% CI, 1.004–1.058; *p* = 0.025). MHD (HR, 1.040 per Gy; *p* = 0.091) and V40 (HR, 1.035 per 1%; *p* = 0.085) showed nonsignificant trends, whereas V50 was not associated with CVAE risk (*p* = 0.304). (Table 3 and Supplementary Table S4)

### 3.4. Overall Survival Analysis

During follow-up, 52 of 86 patients (60.5%) died. Kaplan–Meier analysis demonstrated a median OS of 29 months from RT initiation, with estimated OS rates of 73.1%, 60.1%, 43.9%, and 34.5% at 12, 24, 36, and 60 months, respectively (Figure 4b). In univariable Cox regression, older age (HR, 1.046 per year; 95% CI, 1.013–1.079; *p* = 0.005) and pre-existing cardiovascular comorbidity (HR, 2.728; 95% CI, 1.276–5.832; *p* = 0.010) were associated with increased mortality. In multivariable models adjusted for age, cardiovascular comorbidity, and metastatic status, pre-existing cardiovascular comorbidity demonstrated a consistent association with increased mortality across models (HR range, 2.16–2.45), reaching statistical significance in the V20 (HR, 2.413; 95% CI, 1.038–5.608; *p* = 0.041) and V40 (HR, 2.446; 95% CI, 1.044–5.735; *p* = 0.040) models. None of the evaluated cardiac dosimetric parameters were independently associated with mortality (all *p* > 0.72). Metastatic disease was consistently associated with an approximately twofold higher mortality hazard (HR range, 2.05–2.08), although these associations did not reach statistical significance. Detailed Cox regression analysis is illustrated in Supplementary File S1, Supplementary Tables S5 and S6.

### 3.5. Exploratory Machine Learning Analysis

Four exploratory ML classifiers were evaluated for prediction of CVAEs and overall mortality. Prediction of CVAEs was limited, with discrimination near chance across models, with the highest AUC of 0.57 (95% CI, 0.44–0.69) for GBM. Permutation analysis consistently identified cardiac dosimetric parameters, particularly heart V20 in GBM (mean importance score = 0.140 ± 0.035) and V50 in RF (0.055 ± 0.010), together with age at diagnosis, as the most important clinical predictors, with mean importance scores of 0.028 ± 0.010 and 0.035 ± 0.007 in GBM and RF, respectively. Disease stage also contributed to model predictions, particularly M stage (0.028 ± 0.007) and N stage (0.011 ± 0.004). Feature selection using clinically relevant and higher-importance variables did not materially improve CVAE prediction, with a maximum AUC of 0.54 for GBM, suggesting limited incremental predictive information within this cohort.

Mortality prediction was comparatively better, albeit still modest, in discrimination. In the initial models, the highest performance was by the RF model (AUC = 0.66; 95% CI, 0.53–0.77). Permutation analysis of the RF model identified age at diagnosis (mean AUC decrease, 0.0545 ± 0.0186) and cardiovascular comorbidity (0.0465 ± 0.0200) as the highest-ranked predictors of mortality. Among cardiac dosimetric variables, mean heart dose (0.0257 ± 0.0083) and heart V20 (0.0192 ± 0.0096) showed the greatest predictive contributions. Following feature selection, discrimination improved, with LR achieving the highest AUC of 0.71 (95% CI, 0.59–0.83), followed by RF (AUC, 0.70; 95% CI, 0.57–0.81). Permutation analysis of the reduced-feature models identified MHD as having the greatest importance (mean AUC decrease, 0.1144 ± 0.0352), followed by heart V20 (0.0868 ± 0.0402). Additionally, M stage (0.0678 ± 0.0309), heart V40 (0.0585 ± 0.0323), heart V50 (0.0572 ± 0.0209), age at diagnosis (0.0569 ± 0.0291), and cardiovascular comorbidity (0.0545 ± 0.0252) were among the principal contributors to mortality prediction. Given the small cohort and modest discrimination, these ML findings were considered exploratory and hypothesis-generating rather than clinically predictive. Complete model performance and feature-importance results are provided in Supplementary File S2.

## 4. Discussion

In this real-world cohort of Appalachian patients with lung cancer treated with definitive CRT, cardiovascular morbidity was common and developed across multiple phases of survivorship. CVAEs, including ischemic, pericardial, arrhythmia, and heart failure, were highly common, affecting nearly 60% of patients during treatment and follow-up. Cardiovascular burden accumulated substantially over time during follow-up, with NSTEMI occurring predominantly within the first year of treatment initiation, while pericardial events occurred most frequently within the first two years. Increasing heart V20 and V30 dosimetrics were associated with a higher hazard of CVAE, whereas pre-existing cardiovascular comorbidity, metastatic disease, and V20 were associated with increased overall mortality in this medically vulnerable population. To our knowledge, this is the first study to characterize the burden and spectrum of post-treatment CVAEs in this high-risk Appalachian population while exploring the association of baseline clinical comorbidities and cardiac dosimetric variables with cardiovascular morbidity and mortality. Exploratory ML models demonstrated poor to modest discrimination for CVAE and mortality and, as such, are reported as hypothesis-generating. Age at diagnosis, pre-existing cardiovascular comorbidity, and cardiac radiation dose metrics ranked highest in permutation importance among the variables examined. Collectively, these findings highlight cardiovascular morbidity as an important component of longitudinal care following definitive CRT in an Appalachian lung cancer population.

The high incidence of heterogeneous CVAEs observed in our cohort is notable, reflecting the unique characteristics of the Appalachian population. Appalachia has disproportionately high rates of CVD, tobacco use, and socioeconomic health disparities, factors that may compound the pre-existing cardiovascular risk among patients with lung cancer undergoing multimodality treatment [18,19,20]. Patients with lung cancer frequently have significant smoking histories and pre-existing cardiovascular comorbidities, which may amplify susceptibility to cardiac toxicity following therapy [4,5,15]. In our cohort, 74% of patients had pre-existing cardiovascular comorbidities, 95% were former/current smokers, nearly all had received chemotherapy, and 77% received immunotherapy.

The spectrum of events in this cohort is consistent with prior studies reporting cardiovascular complications after receiving lung cancer treatment [24,25,26]. Dess et al. reported grade ≥ 3 cardiac events in more than 10% of patients with NSCLC treated with RT and identified baseline cardiac disease and higher MHD as associated factors [15]. Niedzielski et al. reported that approximately 50% of patients with stage III NSCLC who were treated with RT developed grade 2 pericardial effusion. However, the CVAEs included in our composite endpoint were pathophysiologically and clinically heterogeneous, encompassing ischemic, pericardial, arrhythmic, and heart-failure-related events. Accordingly, the observed 59% CVAE rate should be interpreted as the overall burden of cardiovascular morbidity following CRT rather than the incidence of a single, uniformly severe or mechanistically homogeneous treatment complication.

Although cardiovascular complications following CRT are multifactorial, cardiac radiation exposure was independently associated with CVAE risk. After adjustment for age and pre-existing cardiovascular comorbidity, heart V20 and V30 were independently associated with time to first CVAE, with each 1-percentage-point increase corresponding to approximately 2.4% and 3.1% higher hazards, respectively. MHD and V40 showed directionally similar but nonsignificant associations. These findings are consistent with the well-established dose-response relationship between cardiac radiation exposure and CVAE risk reported in larger cohorts [12,22,27]. Wang et al. reported two-year symptomatic cardiac event rates of 4%, 7%, and 21% for MHD of <10 Gy, 10–20 Gy, and >20 Gy, respectively, after accounting for baseline CV risk factors [28]. Dess et al. demonstrated that higher MHD was significantly associated with higher cardiac event rates [15]. Current NCCN guidelines therefore recommend heart dose–volume constraints of V40 ≤ 20% and MHD ≤ 20 Gy for RT with concurrent chemotherapy [29]. Heart dosimetric relationships are the focus of ongoing research and clinical trials aimed at establishing standardized organ-at-risk contouring guidelines and dose–volume constraints. However, the observed events cannot be solely attributed to RT. Cardiovascular outcomes following CRT should be interpreted within a multifactorial risk framework. In addition to cardiac radiation exposure, pre-existing CVD, smoking, tumor characteristics (e.g., size, location), and systemic therapies [e.g., platinum-based chemotherapy, immunotherapy, and targeted therapies (osimertinib)] may contribute to both CVAEs and survival [30,31,32,33]. Surgical treatment and other patient-specific factors may further contribute to cardiopulmonary complications and survival outcomes [34]. Therefore, cardiovascular morbidity may reflect a complex interplay among pre-existing diseases, smoking exposure, cancer-related factors, systemic therapy, cardiac radiation exposure, and natural progression of cardiovascular disease. Collectively, these factors highlight the complexity of CVT after CRT and emphasize that radiation dose should be interpreted as one component of a multifactorial risk profile rather than an isolated determinant of outcome.

Overall survival in our study was poor. Older age and pre-existing cardiovascular comorbidity were associated with increased mortality. Cardiovascular comorbidity demonstrated approximately two-fold higher mortality hazard. In contrast, none of the evaluated cardiac dosimetric parameters were independently associated with all-cause mortality. This differs from Chun et al., who reported associations between higher heart V20, V40, and V60 and worse OS [35]. Mortality after definitive CRT for lung cancer is determined by multiple competing factors, including age, cancer stage and biology, treatment response, systemic therapy, and other medical comorbidities [4]. However, our findings may reflect the substantial baseline cardiovascular burden of this medically vulnerable Appalachian population and emphasize that pre-existing cardiovascular health is an important prognostic consideration alongside cancer- and treatment-related factors. Although causality cannot be inferred, these findings highlight the importance of cardiovascular risk assessment and optimization before CRT, with continued surveillance during follow-up, particularly among high-risk patients in Appalachia and underserved areas.

Exploratory ML analyses further illustrate both the potential and current limitations of individualized risk prediction in this setting. CVAE prediction remained poor despite feature selection, indicating that the available clinical and dosimetric variables were insufficient for reliable individual-level CVAE prediction in this small cohort. Mortality models performed somewhat better after feature selection, with the highest AUC of 0.71. Despite their modest discrimination, the exploratory ML models yielded clinically meaningful and biologically plausible findings. Across models, cardiac dosimetry, disease stage, age, and cardiovascular comorbidity ranked among the most influential features for predicting CVAEs or mortality. These findings are broadly consistent with the clinical and survival analyses; however, feature importance does not establish an independent association or causality. Given the small sample size, absence of external validation, and modest discrimination, these models should be considered hypothesis-generating and are not suitable for clinical risk stratification in their current form.

Our study has several important strengths and practical clinical implications for multidisciplinary lung cancer care. In contrast to clinical trial populations which tend to enroll healthier populations with fewer comorbidities, this real-world Appalachian cohort carried a high burden of baseline cardiovascular disease, tobacco exposure, and socioeconomic disparity. Our findings may therefore offer a more representative estimate of cardiovascular risk in this population and inform future cardio-oncology research in routine practice. First, the substantial cardiovascular burden observed before, during, and following definitive CRT supports incorporating cardiovascular risk into multidisciplinary management of patients undergoing definitive CRT for lung cancer, particularly in populations with substantial baseline cardiovascular burden. Identification of high-risk patients before treatment may provide opportunities to optimize modifiable risk factors and, when clinically indicated, involve cardiology or cardio-oncology specialists without delaying oncologic therapy. Second, cardiac radiation exposure should be minimized through adherence to established dose constraints, with target coverage individualized when necessary to maintain treatment safety. In our analysis, higher heart V20 and V30 were independently associated with increased CVAE hazard, supporting attention to the volume of heart receiving low-to-intermediate radiation doses in addition to conventional whole-heart dose assessment. Finally, because CVAEs accumulated throughout follow-up and exhibited different temporal patterns, cardiovascular assessment should not end with completion of CRT. New cardiovascular symptoms, ischemic manifestations, arrhythmias, or relevant abnormalities identified on routine surveillance imaging should prompt appropriate cardiovascular evaluation. These findings support a risk-adapted approach in which patients with substantial baseline cardiovascular risk and/or greater cardiac exposure receive heightened clinical attention for cardiovascular symptoms and appropriate evaluation during survivorship.

Several limitations should be considered when interpreting these findings. The retrospective single-institution design and relatively small sample size limit statistical power and generalizability of the results. Additionally, cardiovascular events were aggregated into a composite outcome despite representing clinically and pathophysiologically heterogeneous conditions with potentially different relationships to cardiac radiation exposure. This approach was used to characterize the overall cardiovascular morbidity burden because the limited number of individual events precluded adequately powered event-specific modeling. Furthermore, standardized severity grading was unavailable because CVAEs were not routinely documented using CTCAE or another standardized grading system in clinical practice; therefore, the composite endpoint reflects the burden and spectrum of cardiovascular morbidity but does not distinguish events according to severity. Consequently, the composite CVAE endpoint should not be interpreted as a uniform toxicity phenotype following CRT, and future larger studies should evaluate individual cardiovascular outcomes separately. Although all CVAEs represented newly developed cardiovascular events occurring during or following definitive CRT, their temporal relationship to treatment does not establish causality. The retrospective design did not permit formal adjudication of whether individual events were attributable to radiation exposure, systemic therapy, baseline cardiovascular risk, cancer-related factors, or other competing causes. Additionally, nearly all patients in this cohort received concurrent chemotherapy, and over three-quarters received immunotherapy, which may confound attribution of CVAE specifically to radiation exposure. Although relevant demographic, cardiovascular, tumor, treatment, and dosimetric factors were evaluated, residual and unmeasured confounding cannot be excluded. Finally, the exploratory ML analyses were further limited by evaluating 20 candidate predictors in only 86 patients, increasing the risk of overfitting and unstable feature importance estimates. Additionally, the ML models underwent internal validation only; therefore, the models do not support individual-level prediction or decision-making, and the identified features require evaluation in larger, externally validated cohorts. External validation in larger independent cohorts will be required before any clinical implementation.

The present study provides real-world evidence to guide future cardio-oncology research in Appalachian and other medically vulnerable populations with lung cancer. Prospective, multi-institutional studies in larger and more diverse cohorts are needed to validate the observed temporal patterns and associations between baseline cardiovascular risk, cardiac radiation exposure, and subsequent CVAEs. Future studies should incorporate cardiac substructure dosimetry, longitudinal multimodality imaging, and circulating biomarkers to improve cardiovascular risk assessment beyond conventional clinical and whole-heart dosimetric measures. Prospective clinical studies are needed to determine whether structured surveillance incorporating echocardiography, biomarkers, ECG monitoring, or cardio-oncology referral improves outcomes and to identify which patients derive the greatest benefit from such strategies. Ultimately, these approaches may help identify high-risk patients, optimize treatment planning, and inform risk-adapted cardiovascular surveillance before, during, and after thoracic RT.

## 5. Conclusions

This study provides the first real-world clinical characterizations of cardiovascular morbidity following definitive CRT in an Appalachian lung cancer population. More than half of the patients experienced a cardiovascular adverse event during or after treatment, with heterogeneous ischemic, pericardial, arrhythmic, and heart failure events accumulating throughout follow-up, highlighting the substantial cardiovascular burden in this high-risk and underserved population. Higher heart V20 and V30 were associated with increased CVAE hazard, while pre-existing cardiovascular comorbidity was an important prognostic factor for overall mortality. Cardiovascular risk assessment and optimization before CRT, attention to cardiac dose during treatment planning, and longitudinal risk-adapted cardiovascular surveillance should be considered important components of multidisciplinary lung cancer care, particularly in medically vulnerable populations. Larger prospective studies are needed to validate these associations and define effective standardized cardiovascular surveillance strategies.

## Figures and Tables

**Table 1:** Descriptive and characteristic table of patients with lung cancer treated with definitive CRT, according to cardiovascular events during or after RT and survival/mortality status.

Variables *n*, (%)	All Patients (*n* = 86)	No CVAE (*n* = 35)	Yes CVAE (*n* = 51)	*p*-Value	Alive (*n* = 34) 40%	Deceased (*n* = 52) 60%	*p*-Value
Age at Diag. Mean, (SD)	66, SD = 9.13	66.5, (SD = 10.05)	65.6, (SD = 8.5)	0.901	62.6, (SD = 8.8)	68.25, (SD = 8.7)	0.004 *
Sex				0.751			0.934
Male	40 (47%)	17 (20%)	23 (27%)		16 (19%)	24 (28%)	
Female	46 (53%)	18 (21%)	28 (33%)		18 (21%)	28 (32%)	
Race				0.564			0.821
White 0	83 (97%)	33 (39%)	50 (58%)		33 (38%)	50 (59%)	
Black 1	3 (3%)	2 (2%)	1 (1%)		1 (1%)	2 (2%)	
Smoking				0.427			0.821
Never	4 (5%)	1 (1%)	3 (3%)		1 (1%)	3 (3%)	
Former	57 (66%)	21 (24%)	36 (42%)		23 (27%)	34 (39%)	
Current	25 (29%)	13 (15%)	12 (14%)		10 (12%)	15 (17%)	
Cardiovascular Comorbidity				0.630			0.008 *
No	22 (25%)	8 (9%)	14 (16%)		14 (16%)	8 (9%)	
Yes	64 (74%)	27 (31%)	37 (43%)		20 (23%)	44 (51%)	
Primary Cancer Type				0.621			0.499
Adenocarcinoma	25 (29%)	11 (13%)	14 (16%)		11 (13%)	14 (16%)	
SCC	39 (45%)	16 (18%)	23 (27%)		14 (16%)	25 (29%)	
SCNEC	17 (20%)	5 (6%)	12 (14%)		8 (9%)	9 (10%)	
LCNEC	1 (1%)	1 (1%)	0 (0%)		0 (0%)	1 (1%)	
NOS	1 (1%)	1 (1%)	0 (0%)		1 (1%)	0 (0%)	
Poorly Differentiated Carcinoma	3 (3%)	1 (1%)	2 (2%)		0 (0%)	3 (3%)	
Site of Primary Tumor				0.221			0.423
Right lung	43 (50%)	21 (24%)	22 (26%)		18 (21%)	25 (29%)	
Left lung	42 (49%)	14 (16%)	28 (33%)		15 (17%)	27 (31%)	
Mediastinal LN with unknown origin	1 (1%)	0 (0%)	1 (1%)		1 (1%)	0 (0%)	
Stage				0.7629			0.5575
Stage I	1 (1%)	0 (0%)	1 (1%)		1 (1%)	0 (0%)	
Stage II	5 (6%)	1 (1%)	4 (5%)		2 (2%)	3 (3%)	
Stage III	57 (66%)	26 (30%)	31 (36%)		22 (25%)	35 (41%)	
Stage IV	6 (7%)	2 (2%)	4 (5%)		1 (1%)	5 (6%)	
Limited Stage (For NEC)	17 (20%)	6 (7%)	11 (13%)		8 (9%)	9 (10%)	
T Stage				0.856			0.343
T1	20 (23%)	9 (10%)	11 (13%)		8 (9%)	12 (14%)	
T2	16 (19%)	6 (7%)	10 (12%)		9 (11%)	7 (8%)	
T3	19 (22%)	6 (12%)	13 (13%)		5 (6%)	14 (16%)	
T4	21 (24%)	10 (11%)	11 (13%)		7 (8%)	14 (16%)	
Tx	4 (5%)	1 (2%)	3 (3%)		3 (3%)	1 (1%)	
NA	6 (7%)	3 (3.5%)	3 (3.5%)		2 (2%)	4 (5%)	
N Stage				0.398			0.613
N0	9 (10%)	3 (3%)	6 (7%)		2 (2%)	7 (8%)	
N1	9 (10%)	5 (6%)	4 (4%)		2 (2%)	7 (8%)	
N2	33 (38%)	15 (17%)	18 (21%)		15 (17%)	18 (21%)	
N3	25 (29%)	7 (8%)	18 (21%)		12 (14%)	13 (15%)	
Nx	4 (5%)	3 (3%)	1 (1%)		1 (1%)	3 (4%)	
NA	6 (7%)	2 (2%)	4 (5%)		2 (2%)	4 (5%)	
M Stage				0.752			0.752
M0/Mx	74 (86%)	30 (35%)	44 (51%)		31 (36%)	43 (50%)	
M1a	0 (0%)	0 (0%)	0 (0%)		0 (0%)	0 (0%)	
M1b	4 (5%)	2 (2.5%)	2 (2.5%)		1 (1%)	3 (4%)	
M1c	2 (2%)	0 (%)	2 (2%)		0 (%)	2 (2%)	
NA	6 (7%)	3 (3.5%)	3 (3.5%)		2 (2%)	4 (5%)	
Immunotherapy				0.942			0.570
No	20 (23%)	8 (9%)	12 (14%)		9 (10%)	11 (13%)	
Yes	66 (77%)	27 (32%)	39 (45%)		25 (29%)	41 (48%)	
Surgery				0.572			0.428
No	74 (86%)	31 (36%)	43 (50%)		28 (33%)	46 (53%)	
Yes	12 (14%)	4 (5%)	8 (9%)		6 (7%)	6 (7%)	

* Indicates statistical significance at *p* < 0.05. Abbreviations: *n*: number; %: percent; SD: standard deviation; NA: not available; SCC: Squamous Cell Carcinoma; SCNEC: Small-Cell Neuroendocrine Carcinoma; LCNEC: Large-Cell Neuroendocrine Carcinoma; NOS: not otherwise specified; NEC: Neuroendocrine Carcinoma.

**Table 2:** Temporal distribution of cardiovascular adverse events by event category.

Time from RT Initiation	Ischemic CVAE (*n* = 15)	Nonischemic CVAE (*n* = 21)	Pericardial-Related CVAE (*n* = 15)
0–6 months	5 (33.3%)	4 (19.0%)	5 (33.3%)
6–12 months	4 (26.7%)	2 (9.5%)	2 (13.3%)
1–2 years	1 (6.7%)	8 (38.1%)	7 (46.7%)
2–5 years	3 (20.0%)	6 (28.6%)	1 (6.7%)
>5 years	2 (13.3%)	1 (4.8%)	0 (0%)

RT: radiation therapy; CVAE: cardiovascular adverse event. Percentages are calculated within each CVAE category. Individual CVAE types were grouped as ischemic, nonischemic, or pericardial-related for temporal analysis. All events that did not fall under ischemic or pericardial-related events were grouped together in the nonischemic CVAE category.

**Table 3:** Multivariable Cox proportional hazards models of time to first cardiovascular adverse event.

Cardiac Exposure	Adjusted HR (95% CI)	*p*-Value	C-Index
Mean heart dose (Gy)	1.040 (0.994–1.089)	0.091	0.599
Heart V20 (%)	1.024 (1.005–1.044)	0.015 *	0.604
Heart V30 (%)	1.031 (1.004–1.058)	0.025 *	0.594
Heart V40 (%)	1.035 (0.995–1.075)	0.085	0.601
Heart V50 (%)	1.030 (0.974–1.089)	0.304	0.593

HR, hazard ratio; CI, confidence interval; C-index, concordance index. Each cardiac dosimetric parameter was evaluated in a separate multivariable Cox proportional hazards model. * Indicates statistical significance at *p* < 0.05.

## Data Availability

The de-identified datasets and analysis code used in this study will be shared upon request based on the institutional requirements.

## Supplementary Materials

The following supporting information can be downloaded at https://www.mdpi.com/article/10.3390/cancers18172830/s1: Supplementary File S1: Supplementary Tables. Supplementary File S2: Tables and figures of machine learning analysis. Supplementary Figures File.

## Abbreviations

The following abbreviations are used in this manuscript:

CVAEs	Cardiovascular Adverse Events
CRT	Chemoradiotherapy
ML	Machine Learning
3D-CRT	Three-Dimensional Conformal Radiotherapy
AI	Artificial Intelligence
AUC	Area Under the Receiver Operating Characteristic Curve
CAD	Coronary Artery Disease
CHF	Congestive Heart Failure
CT	Computed Tomography
CVD	Cardiovascular Disease
CVT	Cardiovascular Toxicity
EHR	Electronic Health Record
GBM	Gradient Boosting Machine
IMRT	Intensity-Modulated Radiation Therapy
IQR	Interquartile Range
LA-NSCLC	Locally Advanced Non-Small-Cell Lung Cancer
LR	Logistic Regression
LS-SCLC	Limited-Stage Small-Cell Lung Cancer
MHD	Mean Heart Dose
NCCN	National Comprehensive Cancer Network
NSCLC	Non-Small-Cell Lung Cancer
NSTEMI	Non-ST-Elevation Myocardial Infarction
OS	Overall Survival
RF	Random Forest
RT	Radiotherapy
CV	Cardiovascular
SD	Standard Deviation
SCLC	Small-Cell Lung Cancer
STEMI	ST-Elevation Myocardial Infarction
SVM	Support Vector Machine
VMAT	Volumetric Modulated Arc Therapy

## References

[1] Siegel R.L., Kratzer T.B., Wagle N.S., Sung H., Jemal A. (2026). Cancer statistics, 2026. CA Cancer J. Clin..

[2] Curran W.J., Paulus R., Langer C.J., Komaki R., Lee J.S., Hauser S., Movsas B., Wasserman T., Rosenthal S.A., Gore E. (2011). Sequential vs. concurrent chemoradiation for stage III non-small cell lung cancer: Randomized phase III trial RTOG 9410. J. Natl. Cancer Inst..

[3] Turrisi A.T., Kim K., Blum R., Sause W.T., Livingston R.B., Komaki R., Wagner H., Aisner S., Johnson D.H. (1999). Twice-daily compared with once-daily thoracic radiotherapy in limited small-cell lung cancer treated concurrently with cisplatin and etoposide. N. Engl. J. Med..

[4] El-Rayes M., Nardi Agmon I., Yu C., Osataphan N., Yu H.A., Hope A., Sacher A., Yu A.F., Abdel-Qadir H., Thavendiranathan P. (2025). Lung Cancer and Cardiovascular Disease: Common Pathophysiology and Treatment-Emergent Toxicity. JACC CardioOncol..

[5] Wilcox N.S., Amit U., Reibel J.B., Berlin E., Howell K., Ky B. (2024). Cardiovascular disease and cancer: Shared risk factors and mechanisms. Nat. Rev. Cardiol..

[6] Verma V., Simone C.B., Werner-Wasik M. (2017). Acute and Late Toxicities of Concurrent Chemoradiotherapy for Locally-Advanced Non-Small Cell Lung Cancer. Cancers.

[7] Verginadis I.I., Citrin D.E., Ky B., Feigenberg S.J., Georgakilas A.G., Hill-Kayser C.E., Koumenis C., Maity A., Bradley J.D., Lin A. (2025). Radiotherapy toxicities: Mechanisms, management, and future directions. Lancet.

[8] Wang K., Tepper J.E. (2021). Radiation therapy-associated toxicity: Etiology, management, and prevention. CA Cancer J. Clin..

[9] Siaravas K.C., Katsouras C.S., Sioka C. (2023). Radiation Treatment Mechanisms of Cardiotoxicity: A Systematic Review. Int. J. Mol. Sci..

[10] Walls G.M., Bergom C., Mitchell J.D., Rentschler S.L., Hugo G.D., Samson P.P., Robinson C.G. (2025). Cardiotoxicity following thoracic radiotherapy for lung cancer. Br. J. Cancer.

[11] Zhao C., Xu S., Yang Y., Shen X., Wang J., Xing S., Yu Z. (2025). Intersection of Cardio-Oncology: An Overview of Radiation-Induced Heart Disease in the Context of Tumors. J. Am. Heart Assoc..

[12] Darby S.C., Ewertz M., McGale P., Bennet A.M., Blom-Goldman U., Bronnum D., Correa C., Cutter D., Gagliardi G., Gigante B. (2013). Risk of ischemic heart disease in women after radiotherapy for breast cancer. N. Engl. J. Med..

[13] Gharzai L., Verma V., Denniston K.A., Bhirud A.R., Bennion N.R., Lin C. (2016). Radiation Therapy and Cardiac Death in Long-Term Survivors of Esophageal Cancer: An Analysis of the Surveillance, Epidemiology, and End Result Database. PLoS ONE.

[14] van Nimwegen F.A., Schaapveld M., Janus C.P., Krol A.D., Petersen E.J., Raemaekers J.M., Kok W.E., Aleman B.M., van Leeuwen F.E. (2015). Cardiovascular disease after Hodgkin lymphoma treatment: 40-year disease risk. JAMA Intern. Med..

[15] Dess R.T., Sun Y., Matuszak M.M., Sun G., Soni P.D., Bazzi L., Murthy V.L., Hearn J.W.D., Kong F.M., Kalemkerian G.P. (2017). Cardiac Events After Radiation Therapy: Combined Analysis of Prospective Multicenter Trials for Locally Advanced Non-Small-Cell Lung Cancer. J. Clin. Oncol..

[16] Locquet M., Jacob S., Geets X., Beaudart C. (2024). Dose-volume predictors of cardiac adverse events after high-dose thoracic radiation therapy for lung cancer: A systematic review and meta-analysis. BMC Cancer.

[17] Watanabe Y., Koide Y., Shimizu H., Aoyama T., Shindo Y., Hashimoto S., Tachibana H., Kodaira T. (2024). Risk Stratification by Combination of Heart and Lung Dose in Locally Advanced Non-Small-Cell Lung Cancer after Radiotherapy. Cancers.

[18] Roth G.A., Johnson C.O., Abate K.H., Abd-Allah F., Ahmed M., Alam K., Alam T., Alvis-Guzman N., Ansari H., Global Burden of Cardiovascular Diseases Collaboration (2018). The Burden of Cardiovascular Diseases Among US States, 1990–2016. JAMA Cardiol..

[19] Roth G.A., Dwyer-Lindgren L., Bertozzi-Villa A., Stubbs R.W., Morozoff C., Naghavi M., Mokdad A.H., Murray C.J.L. (2017). Trends and Patterns of Geographic Variation in Cardiovascular Mortality Among US Counties, 1980–2014. JAMA.

[20] American Cancer Society (2026). Cancer Prevention & Early Detection Facts & Figures Tables and Figures 2026.

[21] Qiao E.M., He J., Silos K.D., Gasho J.O., Belen P., Bitterman D.S., McKenzie E., Steers J., Guthier C., Nohria A. (2025). Baseline atrial volume indices and major adverse cardiac events following thoracic radiotherapy. Front. Cardiovasc. Med..

[22] Yoo S.K., Kim K.H., Noh J.M., Oh J., Yang G., Kim J., Kim N., Kim H., Yoon H.I. (2024). Development of learning-based predictive models for radiation-induced atrial fibrillation in non-small cell lung cancer patients by integrating patient-specific clinical, dosimetry, and diagnostic information. Radiother. Oncol..

[23] Salama V., Godinich B.M., Dunham N., Nguyen T., Wen S., Schmidlen J.A., Cox W., Lilly P.M., Ryckman J., Siochi R.A. (2026). A systematic review of artificial intelligence in radiotherapy associated cardiovascular toxicity. Cardiooncology.

[24] Banfill K., Giuliani M., Aznar M., Franks K., McWilliam A., Schmitt M., Sun F., Vozenin M.C., Faivre Finn C., Committee IART (2021). Cardiac Toxicity of Thoracic Radiotherapy: Existing Evidence and Future Directions. J. Thorac. Oncol..

[25] Hufnagle J.J., Andersen S.N., Maani E.V. (2025). Radiation-Induced Cardiac Toxicity.

[26] Belzile-Dugas E., Eisenberg M.J. (2021). Radiation-Induced Cardiovascular Disease: Review of an Underrecognized Pathology. J. Am. Heart Assoc..

[27] Speirs C.K., DeWees T.A., Rehman S., Molotievschi A., Velez M.A., Mullen D., Fergus S., Trovo M., Bradley J.D., Robinson C.G. (2017). Heart Dose Is an Independent Dosimetric Predictor of Overall Survival in Locally Advanced Non-Small Cell Lung Cancer. J. Thorac. Oncol..

[28] Wang K., Eblan M.J., Deal A.M., Lipner M., Zagar T.M., Wang Y., Mavroidis P., Lee C.B., Jensen B.C., Rosenman J.G. (2017). Cardiac Toxicity After Radiotherapy for Stage III Non-Small-Cell Lung Cancer: Pooled Analysis of Dose-Escalation Trials Delivering 70 to 90 Gy. J. Clin. Oncol..

[29] NCCN National Comprehensive Cancer Network (2025). NCCN Clinical Practice Guidelines in Oncology: Non-Small Cell Lung Cancer. https://www.nccn.org/guidelines/guidelines-detail?category=1&id=1450.

[30] Childs B.G., Durik M., Baker D.J., van Deursen J.M. (2015). Cellular senescence in aging and age-related disease: From mechanisms to therapy. Nat. Med..

[31] Herrmann J., Yang E.H., Iliescu C.A., Cilingiroglu M., Charitakis K., Hakeem A., Toutouzas K., Leesar M.A., Grines C.L., Marmagkiolis K. (2016). Vascular Toxicities of Cancer Therapies: The Old and the New—An Evolving Avenue. Circulation.

[32] Patel N.P., Dressler D., Zhu M., Liu Y., Sun Z., Patel J.D. (2024). Predictive factors for cardiac events following concurrent chemotherapy and radiation with durvalumab consolidation for unresectable non-metastatic non-small cell lung cancer. J. Clin. Oncol..

[33] Agbarya A., Raphael A., Gantz Sorotsky H., Rottenberg Y., Sebek V., Radonjic D., Yakobson A., Arnon J., Shalata W. (2025). Real-World Data on Osimertinib-Associated Cardiac Toxicity. J. Clin. Med..

[34] Lugg S.T., Agostini P.J., Tikka T., Kerr A., Adams K., Bishay E., Kalkat M.S., Steyn R.S., Rajesh P.B., Thickett D.R. (2016). Long-term impact of developing a postoperative pulmonary complication after lung surgery. Thorax.

[35] Chun S.G., Hu C., Choy H., Komaki R.U., Timmerman R.D., Schild S.E., Bogart J.A., Dobelbower M.C., Bosch W., Galvin J.M. (2017). Impact of Intensity-Modulated Radiation Therapy Technique for Locally Advanced Non-Small-Cell Lung Cancer: A Secondary Analysis of the NRG Oncology RTOG 0617 Randomized Clinical Trial. J. Clin. Oncol..

