# Supplementary material for: Cardiovascular Morbidity Following Definitive Chemoradiotherapy for Lung Cancer in an Appalachian Population
Source: Cancers (Basel). 2026 Sep 1;18(17):2830. doi: 10.3390/cancers18172830 (PMC13565158; doi:10.3390/cancers18172830)
Supplement: Supplementary file 1 [file cancers-18-02830-s001.zip › Supplementary Figures.docx]

**Supplementary Figures**

Supplementary Figure S1

Supplementary Figure S1: Correlation matrix of whole-heart dosimetric parameters. Spearman correlation coefficients (rₛ) are shown for mean heart dose (MHD) and heart V20, V30, V40, and V50. Strong positive correlations were observed among the cardiac dosimetric parameters, with correlation coefficients ranging from 0.70 to 0.96, indicating substantial intercorrelation among whole-heart dose metrics. This collinearity was considered in subsequent statistical analyses by evaluating individual cardiac dosimetric parameters in separate multivariable Cox regression models.
