# Supplementary material for: Cardiovascular Morbidity Following Definitive Chemoradiotherapy for Lung Cancer in an Appalachian Population
Source: Cancers (Basel). 2026 Sep 1;18(17):2830. doi: 10.3390/cancers18172830 (PMC13565158; doi:10.3390/cancers18172830)
Supplement: Supplementary file 1 [file cancers-18-02830-s001.zip › Supplementary File S1_Supplementary Tables.docx]

**Supplementary Tables**

**Supplementary Table S1.** Characteristic table of dosimetrics among patients with lung cancers and treated with CRT, according to cardiovascular events during or after RT and survival/mortality status.

| Variables | All Patients (n = 86) | No CVAE (n = 35) | Yes CVAE (n = 51) | P value | Alive (n = 34) 40% | Deceased (n = 52) 60% | P value |
| --- | --- | --- | --- | --- | --- | --- | --- |
| Mean Heart (Gy) | Median=(11.8), IQR=5.70-14.88 | Median=9.44, IQR=6.34-14.69 | Median=13.38, IQR=5.56-15.48 | 0.272 | Median=10.23, IQR=7.51-14.36 | Median=12.98, IQR=4.71-16.12 | 0.937 |
| Heart V50 (%) | Median=3.72,  IQR=0.03-10.73 | Median=1.36, IQR=0.24-5.51 | Median=1.4, IQR=0-5.95 | 0.671 | Median=1.32, IQR=0.18-5.19 | Median=1.41, IQR=0.02-5.88 | 0.919 |
| Heart V40 (%) | Median=3.72,  IQR=1.03-10.73 | Median=3.69,  IQR=1.13-9.96 | Median=4.04, IQR=0.90-12.56 | 0.754 | Median=2.73,  IQR=1.01-10.51 | Median=4.06, IQR=1.04-10.83 | 0.708 |

**Supplementary Table S2.** Temporal Distribution of Cardiovascular Adverse Events by Event Type

| **Time from RT Initiation** | **NSTEMI**  **(n=15)** | **Pericardial Disease**  **(n=15)** | **Arrhythmia**  **(n=8)** | **Congestive Heart Failure**  **(n=4)** | **Valvular Dysfunction**  **(n=2)** | **Cardiology Referral**  **(n=4)** | **Other***  **(n=3)** |
| --- | --- | --- | --- | --- | --- | --- | --- |
| **0-6 months** | 5 (33.3%) | 5 (33.3%) | 2 (25.0%) | 0 (0%) | 0 (0%) | 2 (50.0%) | 0 (0%) |
| **6-12 months** | 4 (26.7%) | 2 (13.3%) | 1 (12.5%) | 1 (25.0%) | 0 (0%) | 0 (0%) | 1 (33.3%) |
| **1-2 years** | 1 (6.7%) | 7 (46.7%) | 2 (25.0%) | 1 (25.0%) | 1 (50%) | 1 (25.0%) | 2 (66.7%) |
| **2-5 years** | 3 (20.0%) | 1 (6.7%) | 3 (37.5%) | 1 (25.0%) | 1 (50%) | 1 (25.0%) | 0 (0%) |
| **>5 years** | 2 (13.3%) | 0 (0%) | 0 (0%) | 1 (25.0%) | 0 (0%) | 0 (0%) | 0 (0%) |

*RT = radiation therapy; CVAE = cardiovascular adverse event*

*Percentages are calculated within each CVAE category.*

**Other events include: LV apical aneurysm at 1-2 years, cardiomyopathy at 1-2 years, reduced EF at 6-12 months*

**Supplementary Table S3.** Univariable Cox Proportional Hazards Regression Analysis of Time From Radiotherapy Initiation to First Cardiovascular Adverse Event.

| **Variable** | **Comparison** | **HR** | **CI_lower** | **CI_upper** | **P_value** |
| --- | --- | --- | --- | --- | --- |
| **M Stage** | M Stage_4.0 | 4.503 | 1.047 | 19.373 | 0.0433 |
| **Site of Primary** | Site of Primary_1 | 1.753 | 0.986 | 3.118 | 0.056 |
| **Heart V20 (%)** | Per 1-unit increase | 1.017 | 0.998 | 1.036 | 0.0734 |
| **Tumor_Histology** | Tumor_Histology_6 | 3.647 | 0.79 | 16.836 | 0.0973 |
| **Heart V30 (%)** | Per 1-unit increase | 1.02 | 0.995 | 1.046 | 0.125 |
| **Tumor_Histology** | Tumor_Histology_3 | 1.818 | 0.835 | 3.96 | 0.1321 |
| **Cardiovascular comorbidity** | Cardiovascular comorbidity_1 | 1.592 | 0.841 | 3.012 | 0.1534 |
| **N Stage** | N Stage_5.0 | 0.232 | 0.028 | 1.947 | 0.1782 |
| **Mean Heart (Gy)** | Per 1-unit increase | 1.028 | 0.983 | 1.075 | 0.22 |
| **RT1 Fractions** | Per 1-unit increase | 1.099 | 0.942 | 1.281 | 0.2309 |
| **N Stage** | N Stage_3.0 | 0.585 | 0.23 | 1.484 | 0.2589 |
| **Stage at Dx** | Stage at Dx_4 | 3.487 | 0.366 | 33.177 | 0.2772 |
| **Heart V40 (%)** | Per 1-unit increase | 1.02 | 0.983 | 1.058 | 0.3021 |
| **Smoking** | Smoking_1 | 1.811 | 0.54 | 6.071 | 0.336 |
| **Age_at_Diagnosis** | Per 1-unit increase | 1.016 | 0.983 | 1.049 | 0.3456 |
| **Stage at Dx** | Stage at Dx_5 | 2.523 | 0.309 | 20.592 | 0.3877 |
| **Surgery?** | Surgery?_1 | 0.717 | 0.331 | 1.555 | 0.3999 |
| **T Stage** | T Stage_5.0 | 0.612 | 0.162 | 2.317 | 0.4696 |
| **Stage at Dx** | Stage at Dx_2 | 2.037 | 0.219 | 18.909 | 0.5314 |
| **T Stage** | T Stage_2.0 | 0.806 | 0.338 | 1.92 | 0.6267 |
| **Heart V50 (%)** | Per 1-unit increase | 1.012 | 0.96 | 1.068 | 0.653 |
| **Site of Primary** | Site of Primary_3 | 1.575 | 0.21 | 11.825 | 0.6589 |
| **T Stage** | T Stage_3.0 | 1.196 | 0.531 | 2.694 | 0.665 |
| **Race_Ethnicity** | Race_Ethnicity_1 | 0.681 | 0.094 | 4.961 | 0.7047 |
| **M Stage** | M Stage_3.0 | 1.311 | 0.313 | 5.497 | 0.7113 |
| **Stage at Dx** | Stage at Dx_3 | 1.456 | 0.191 | 11.084 | 0.7165 |
| **RT1 Dose (in Gy)** | Per 1-unit increase | 1.008 | 0.962 | 1.057 | 0.7272 |
| **Sex** | Sex_1 | 0.921 | 0.529 | 1.603 | 0.7719 |
| **Immunotherapy?** | Immunotherapy?_1 | 1.092 | 0.556 | 2.147 | 0.7977 |
| **M Stage** | M Stage_5.0 | 0.894 | 0.374 | 2.133 | 0.7999 |
| **Tumor_Histology** | Tumor_Histology_1 | 1.075 | 0.542 | 2.132 | 0.8354 |
| **N Stage** | N Stage_4.0 | 0.927 | 0.365 | 2.354 | 0.8738 |
| **T Stage** | T Stage_4.0 | 0.963 | 0.41 | 2.258 | 0.9305 |
| **Smoking** | Smoking_2 | 1.034 | 0.284 | 3.77 | 0.9596 |
| **N Stage** | N Stage_2.0 | 1.017 | 0.285 | 3.632 | 0.9787 |
| **Tumor_Histology** | Tumor_Histology_5 | 0 | 0 | inf | 0.9968 |
| **Tumor_Histology** | Tumor_Histology_4 | 0 | 0 | inf | 0.9975 |

*Abbreviations: CI, confidence interval; CVAE, cardiovascular adverse event; HR, hazard ratio; RT, radiation therapy. Time to CVAE was calculated from RT initiation to the first documented CVAE. Patients without a CVAE were censored at their last available follow-up. HRs for continuous variables represent the change in hazard associated with a 1-unit increase (1 year for age, 1 Gy for radiation dose, and 1 percentage point for heart V20–V50). For categorical variables, HRs are reported relative to the corresponding reference category. Sample size and number of CVAEs vary because of missing data for individual predictors. Two-sided P<.05 was considered statistically significant.*

**Supplementary Table S4.** Multivariable Cox Proportional Hazards Regression Models Analysis of Time From Radiotherapy Initiation to First Cardiovascular Adverse Event.

| **Model** | **Variable** | **Adjusted HR** | **95% CI Lower** | **95% CI Upper** | **P Value** | **Concordance** | **Adjusted HR (95% CI)** |
| --- | --- | --- | --- | --- | --- | --- | --- |
| V20 Model | Heart V20 (%) | 1.024 | 1.005 | 1.044 | 0.0153 | 0.604 | 1.024 (1.005-1.044) |
| V30 Model | Heart V30 (%) | 1.031 | 1.004 | 1.058 | 0.0254 | 0.594 | 1.031 (1.004-1.058) |
| V20 Model | M_stage_4 | 5.246 | 1.2 | 22.933 | 0.0276 | 0.604 | 5.246 (1.200-22.933) |
| MHD Model | M_stage_4 | 4.965 | 1.134 | 21.744 | 0.0334 | 0.599 | 4.965 (1.134-21.744) |
| V30 Model | M_stage_4 | 4.773 | 1.102 | 20.67 | 0.0366 | 0.594 | 4.773 (1.102-20.670) |
| V40 Model | M_stage_4 | 4.59 | 1.063 | 19.822 | 0.0412 | 0.601 | 4.590 (1.063-19.822) |
| V50 Model | M_stage_4 | 4.249 | 0.99 | 18.234 | 0.0516 | 0.593 | 4.249 (0.990-18.234) |
| Core Clinical Model | M_stage_4 | 4.114 | 0.961 | 17.612 | 0.0566 | 0.591 | 4.114 (0.961-17.612) |
| V40 Model | Heart V40 (%) | 1.035 | 0.995 | 1.075 | 0.0853 | 0.601 | 1.035 (0.995-1.075) |
| V30 Model | Cardiovascular comorbidity | 1.876 | 0.909 | 3.872 | 0.0888 | 0.594 | 1.876 (0.909-3.872) |
| MHD Model | Mean Heart (Gy) | 1.04 | 0.994 | 1.089 | 0.0911 | 0.599 | 1.040 (0.994-1.089) |
| V20 Model | Cardiovascular comorbidity | 1.733 | 0.851 | 3.527 | 0.1297 | 0.604 | 1.733 (0.851-3.527) |
| V40 Model | Cardiovascular comorbidity | 1.702 | 0.819 | 3.537 | 0.1543 | 0.601 | 1.702 (0.819-3.537) |
| V50 Model | Cardiovascular comorbidity | 1.6 | 0.786 | 3.253 | 0.1948 | 0.593 | 1.600 (0.786-3.253) |
| MHD Model | Cardiovascular comorbidity | 1.548 | 0.782 | 3.064 | 0.2096 | 0.599 | 1.548 (0.782-3.064) |
| Core Clinical Model | Cardiovascular comorbidity | 1.463 | 0.741 | 2.888 | 0.2727 | 0.591 | 1.463 (0.741-2.888) |
| V50 Model | Heart V50 (%) | 1.03 | 0.974 | 1.089 | 0.3043 | 0.593 | 1.030 (0.974-1.089) |
| V20 Model | Age_at_Diagnosis | 1.013 | 0.978 | 1.05 | 0.4633 | 0.604 | 1.013 (0.978-1.050) |
| V40 Model | Age_at_Diagnosis | 1.012 | 0.977 | 1.048 | 0.4986 | 0.601 | 1.012 (0.977-1.048) |
| MHD Model | Age_at_Diagnosis | 1.012 | 0.977 | 1.048 | 0.5052 | 0.599 | 1.012 (0.977-1.048) |
| V50 Model | Age_at_Diagnosis | 1.01 | 0.975 | 1.045 | 0.5858 | 0.593 | 1.010 (0.975-1.045) |
| Core Clinical Model | Age_at_Diagnosis | 1.008 | 0.974 | 1.044 | 0.6369 | 0.591 | 1.008 (0.974-1.044) |
| V30 Model | Age_at_Diagnosis | 1.006 | 0.972 | 1.041 | 0.7384 | 0.594 | 1.006 (0.972-1.041) |

*Footnote: Multivariable Cox proportional hazards regression models evaluated time from radiotherapy initiation to the first documented cardiovascular adverse event (CVAE). The core clinical model included age at diagnosis, pre-existing cardiovascular comorbidity, and the M-stage category identified in univariable analysis. Because of substantial intercorrelation among whole-heart dosimetric parameters, mean heart dose (MHD), heart V20, V30, V40, and V50 were entered separately into individual adjusted models rather than simultaneously. Adjusted hazard ratios (HRs) for continuous dosimetric variables represent the change in CVAE hazard per 1-Gy increase in MHD or per 1-percentage-point increase in heart V20–V50; age was modeled per 1-year increase. Concordance represents the Harrell C-index for each model. Two-sided P<0.05 was considered statistically significant. Abbreviations: CI, confidence interval; CVAE, cardiovascular adverse event; HR, hazard ratio; MHD, mean heart dose; RT, radiation therapy; V20–V50, percentage of the heart volume receiving ≥20–50 Gy, respectively.*

**Supplementary Table S5.** Univariable Cox Proportional Hazards Regression Analysis of Time From Radiotherapy Initiation to Mortality.

| **Variable** | **HR** | **CI_lower** | **CI_upper** | **P_value** |
| --- | --- | --- | --- | --- |
| Age_at_Diagnosis | 1.046 | 1.013 | 1.079 | 0.0053 |
| Cardiovascular comorbidity | 2.728 | 1.276 | 5.832 | 0.0096 |
| Tumor_Histology | 2.998 | 0.85 | 10.571 | 0.0877 |
| M_stage_binary | 2.082 | 0.818 | 5.296 | 0.1238 |
| T Stage | 0.273 | 0.035 | 2.133 | 0.2157 |
| N Stage | 0.577 | 0.24 | 1.388 | 0.2192 |
| Surgery? | 0.602 | 0.255 | 1.421 | 0.2467 |
| N Stage | 0.593 | 0.236 | 1.49 | 0.266 |
| N Stage | 1.762 | 0.615 | 5.049 | 0.2914 |
| T Stage | 0.613 | 0.24 | 1.562 | 0.3049 |
| RT1 Dose (in Gy) | 1.025 | 0.978 | 1.073 | 0.3055 |
| Tumor_Histology | 1.374 | 0.714 | 2.644 | 0.3421 |
| RT1 Fractions | 1.051 | 0.912 | 1.211 | 0.4939 |
| T Stage | 1.297 | 0.598 | 2.813 | 0.5111 |
| Sex | 0.859 | 0.496 | 1.49 | 0.5898 |
| Tumor_Histology | 1.539 | 0.201 | 11.752 | 0.6778 |
| Site of Primary | 1.108 | 0.643 | 1.912 | 0.7112 |
| Smoking | 1.253 | 0.362 | 4.343 | 0.722 |
| Heart V20 (%) | 1.003 | 0.987 | 1.019 | 0.7306 |
| Smoking | 1.229 | 0.376 | 4.018 | 0.7333 |
| Immunotherapy? | 1.116 | 0.568 | 2.191 | 0.7502 |
| Race_Ethnicity | 1.211 | 0.294 | 4.993 | 0.7909 |
| Heart V50 (%) | 0.995 | 0.942 | 1.05 | 0.8427 |
| Tumor_Histology | 1.077 | 0.465 | 2.493 | 0.8633 |
| Heart V30 (%) | 1.002 | 0.979 | 1.025 | 0.893 |
| N Stage | 0.913 | 0.235 | 3.539 | 0.8951 |
| Heart V40 (%) | 1.002 | 0.967 | 1.038 | 0.9271 |
| Mean Heart (Gy) | 1 | 0.962 | 1.04 | 0.9944 |
| Stage at Dx | 15956078 | 0 | inf | 0.9953 |
| Stage at Dx | 8277431 | 0 | inf | 0.9955 |
| Stage at Dx | 7830839 | 0 | inf | 0.9955 |
| Stage at Dx | 7146890 | 0 | inf | 0.9955 |
| Tumor_Histology | 0 | 0 | inf | 0.9956 |
| T Stage | 1.002 | 0.457 | 2.197 | 0.9958 |
| Site of Primary | 0 | 0 | inf | 0.9959 |

*Footnote: Univariable Cox proportional hazards regression was used to evaluate associations between individual clinical, treatment, and cardiac dosimetric variables and all-cause mortality. Survival time was calculated from RT initiation to death or last available follow-up, with patients alive at last follow-up censored. Hazard ratios (HRs) for continuous variables represent the change in mortality hazard per 1-year increase in age, per 1-Gy increase in radiation dose or mean heart dose, and per 1-percentage-point increase in heart V20–V50. For categorical variables, HRs are reported relative to the corresponding reference category. Two-sided P<.05 was considered statistically significant. Abbreviations: CI, confidence interval; HR, hazard ratio; RT, radiation therapy; V20–V50, percentage of the heart volume receiving ≥20–50 Gy, respectively.*

**Supplementary Table S6.** Multivariable Cox Proportional Hazards Regression Models Analysis of Time From Radiotherapy Initiation to Mortality.

| **Model** | **Variable** | **Deaths** | **Adjusted HR** | **95% CI Lower** | **95% CI Upper** | **Adjusted HR (95% CI)** | **P Value** | **Concordance** |
| --- | --- | --- | --- | --- | --- | --- | --- | --- |
| V40 Model | Cardiovascular comorbidity | 48 | 2.446 | 1.044 | 5.735 | 2.446 (1.044-5.735) | 0.0396 | 0.615 |
| V20 Model | Cardiovascular comorbidity | 48 | 2.413 | 1.038 | 5.608 | 2.413 (1.038-5.608) | 0.0407 | 0.608 |
| V50 Model | Cardiovascular comorbidity | 48 | 2.206 | 0.954 | 5.101 | 2.206 (0.954-5.101) | 0.0642 | 0.624 |
| MHD Model | Cardiovascular comorbidity | 48 | 2.163 | 0.945 | 4.955 | 2.163 (0.945-4.955) | 0.068 | 0.614 |
| V30 Model | Cardiovascular comorbidity | 48 | 2.167 | 0.942 | 4.985 | 2.167 (0.942-4.985) | 0.0688 | 0.616 |
| V50 Model | M_stage_binary | 48 | 2.076 | 0.812 | 5.308 | 2.076 (0.812-5.308) | 0.1272 | 0.624 |
| MHD Model | Age_at_Diagnosis | 48 | 1.028 | 0.992 | 1.066 | 1.028 (0.992-1.066) | 0.1328 | 0.614 |
| V40 Model | M_stage_binary | 48 | 2.062 | 0.802 | 5.299 | 2.062 (0.802-5.299) | 0.1329 | 0.615 |
| V20 Model | M_stage_binary | 48 | 2.082 | 0.8 | 5.421 | 2.082 (0.800-5.421) | 0.133 | 0.608 |
| V30 Model | Age_at_Diagnosis | 48 | 1.028 | 0.991 | 1.066 | 1.028 (0.991-1.066) | 0.1366 | 0.616 |
| V30 Model | M_stage_binary | 48 | 2.052 | 0.793 | 5.308 | 2.052 (0.793-5.308) | 0.1382 | 0.616 |
| V50 Model | Age_at_Diagnosis | 48 | 1.028 | 0.991 | 1.066 | 1.028 (0.991-1.066) | 0.141 | 0.624 |
| MHD Model | M_stage_binary | 48 | 2.057 | 0.78 | 5.423 | 2.057 (0.780-5.423) | 0.1447 | 0.614 |
| V20 Model | Age_at_Diagnosis | 48 | 1.021 | 0.984 | 1.06 | 1.021 (0.984-1.060) | 0.2743 | 0.608 |
| V40 Model | Age_at_Diagnosis | 48 | 1.021 | 0.983 | 1.06 | 1.021 (0.983-1.060) | 0.2862 | 0.615 |
| V50 Model | Heart V50 (%) | 48 | 1.011 | 0.954 | 1.071 | 1.011 (0.954-1.071) | 0.7235 | 0.624 |
| V40 Model | Heart V40 (%) | 48 | 1.006 | 0.968 | 1.047 | 1.006 (0.968-1.047) | 0.7541 | 0.615 |
| V20 Model | Heart V20 (%) | 48 | 1.003 | 0.986 | 1.019 | 1.003 (0.986-1.019) | 0.7696 | 0.608 |
| MHD Model | Mean Heart (Gy) | 48 | 1.001 | 0.961 | 1.043 | 1.001 (0.961-1.043) | 0.965 | 0.614 |
| V30 Model | Heart V30 (%) | 48 | 1 | 0.976 | 1.025 | 1.000 (0.976-1.025) | 0.9709 | 0.616 |

*Footnote: Multivariable Cox proportional hazards regression models evaluated factors associated with all-cause mortality following definitive CRT. Each model was adjusted for age at diagnosis, pre-existing cardiovascular comorbidity, and M stage. Because of substantial intercorrelation among whole-heart dosimetric parameters, mean heart dose (MHD), heart V20, V30, V40, and V50 were evaluated separately in individual multivariable models rather than entered simultaneously. “M stage was dichotomized as M0/Mx versus M1. Adjusted HRs for continuous variables represent the change in mortality hazard per 1-year increase in age, per 1-Gy increase in MHD, and per 1-percentage-point increase in heart V20–V50. Concordance represents the Harrell C-index for each model. Two-sided P<0.05 was considered statistically significant. Abbreviations: CI, confidence interval; CRT, chemoradiotherapy; HR, hazard ratio; MHD, mean heart dose; RT, radiation therapy; V20–V50, percentage of the heart volume receiving ≥20–50 Gy, respectively.*
