# Supplementary material for: Cardiovascular Morbidity Following Definitive Chemoradiotherapy for Lung Cancer in an Appalachian Population
Source: Cancers (Basel). 2026 Sep 1;18(17):2830. doi: 10.3390/cancers18172830 (PMC13565158; doi:10.3390/cancers18172830)
Supplement: Supplementary file 1 [file cancers-18-02830-s001.zip › Supplementary File S2_ML Tables and Figures.docx]

**Supplementary Tables and Figures of Machine Learning Models Analysis.**

| Model | AUC, 95% CI | Sensitivity, 95% CI | Specificity, 95% CI | F1, 95% CI |
| --- | --- | --- | --- | --- |
| Gradient Boosting Machine (GBM) | 0.567, [0.440, 0.686] | 0.725, [0.596, 0.846] | 0.286, [0.139, 0.438] | 0.655, [0.542, 0.752] |
| Logistic Regression (LR) | 0.524, [0.397, 0.646] | 0.588, [0.444, 0.727] | 0.457, [0.294, 0.622] | 0.600, [0.469, 0.706] |
| Random Forest Model (RF) | 0.514, [0.381, 0.639] | 0.804, [0.689, 0.909] | 0.171, [0.056, 0.297] | 0.678, [0.579, 0.768] |
| Support Vector Machine (SVM) | 0.492, [0.360, 0.622] | 0.725, [0.600, 0.840] | 0.314, [0.167, 0.485] | 0.661, [0.552, 0.759] |

**ML Supplementary Table S1:** Discrimination metrics and the performance of the models predicting CVAE during and after CRT in Appalachian lung cancer population.

(a)

(b)

**ML Supplementary Figure S1:** Feature importance of tree-based ML models predicting CVAEs during and after definitive CRT in lung cancer population using permutation feature importance. (a) Feature importance of the top 15 features of Gradient Boosting Model. (b) Feature importance of the top 15 features of Random Forest Model.

**ML Supplementary Table S2:** Feature importance of Random Forest model predicting Cardiovascular adverse events (CVAEs) in lung cancer during or after chemoradiation.

| Feature | Importance_mean | Importance_std |
| --- | --- | --- |
| num__Heart V50 (%) | 0.054827 | 0.009584 |
| num__Age_at_Diagnosis | 0.035294 | 0.006659 |
| num__Heart V20 (%) | 0.028273 | 0.017337 |
| cat__M Stage | 0.027768 | 0.006962 |
| num__Mean Heart (Gy) | 0.023137 | 0.019315 |
| num__Heart V40 (%) | 0.022988 | 0.006574 |
| cat__Site of Primary | 0.016564 | 0.006655 |
| cat__N Stage | 0.011092 | 0.003925 |
| cat__Smoking | 0.008571 | 0.004572 |
| num__Heart V30 (%) | 0.00788 | 0.003152 |
| cat__T Stage | 0.006947 | 0.002981 |
| cat__Tumor_Histology | 0.00648 | 0.002239 |
| cat__Stage at Dx | 0.003978 | 0.002418 |
| cat__Sex | 0.002017 | 0.001291 |
| cat__Immunotherapy? | 0.000878 | 0.000761 |
| num__RT1 Dose (in Gy) | 0.000392 | 0.00165 |
| cat__Surgery? | 0.000317 | 0.000625 |
| cat__Race_Ethnicity | 0 | 0 |
| cat__Cardiovascular comorbidity | -0.0003 | 0.000967 |
| num__RT1 Fractions | -0.00131 | 0.001213 |

**ML Supplementary Table S3:** Feature importance of Gradient Boosting model predicting Cardiovascular adverse events (CVAEs) in lung cancer during or after chemoradiation.

| Feature | Importance_mean | Importance_std |
| --- | --- | --- |
| num__Heart V20 (%) | 0.140411 | 0.035114 |
| num__Age_at_Diagnosis | 0.028422 | 0.010304 |
| num__Heart V40 (%) | 0.010943 | 0.005886 |
| num__Mean Heart (Gy) | 0.007656 | 0.005362 |
| num__Heart V30 (%) | 0.003959 | 0.002893 |
| cat__Site of Primary | 0.001662 | 0.001471 |
| num__RT1 Fractions | 0 | 0 |
| num__RT1 Dose (in Gy) | 0 | 0 |
| cat__Sex | 0 | 0 |
| cat__Race_Ethnicity | 0 | 0 |
| cat__Smoking | 0 | 0 |
| num__Heart V50 (%) | 0 | 0 |
| cat__Cardiovascular comorbidity | 0 | 0 |
| cat__Tumor_Histology | 0 | 0 |
| cat__Stage at Dx | 0 | 0 |
| cat__T Stage | 0 | 0 |
| cat__N Stage | 0 | 0 |
| cat__M Stage | 0 | 0 |
| cat__Immunotherapy? | 0 | 0 |
| cat__Surgery? | 0 | 0 |

**ML Supplementary Table S4:** Discrimination metrics and the performance of the models predicting mortality after CRT in Appalachian lung cancer population.

| **Model** | **AUC, 95% CI** | **Sensitivity, 95% CI** | **Specificity, 95% CI** | **F1, 95% CI** |
| --- | --- | --- | --- | --- |
| Random Forest Model (RF) | 0.658, [0.531, 0.774] | 0.769, [0.649, 0.878] | 0.50, [0.333, 0.667] | 0.734, [0.63, 0.822] |
| Logistic Regression (LR) | 0.644, [0.510, 0.712] | 0.712, [0.583, 0.830] | 0.529, [0.353, 0.697] | 0.705, [0.596, 0.796] |
| Support Vector Machine (SVM) | 0.636, [0.513, 0.756] | 0.827, [0.717, 0.926] | 0.529, [0.367, 0.690] | 0.775, [0.68, 0.852] |
| Gradient Boosting Machine (GBM) | 0.61, [0.483, 0.734] | 0.692, [0.562, 0.815] | 0,529 [0.371, 0.692] | 0.692, [0.582, 0.784] |

**ML Supplementary Figure S2:** Feature importance of the top 15 features of Random Forest Model predicting mortality during and after definitive CRT in lung cancer population using permutation feature importance.

**ML Supplementary Table S5:** Feature importance of Random Forest model predicting mortality in lung cancer during or after chemoradiation.

| Variable | Mean AUC Decrease | SD |
| --- | --- | --- |
| Age_at_Diagnosis | 0.0545 | 0.0186 |
| Cardiovascular comorbidity | 0.0465 | 0.02 |
| Mean Heart (Gy) | 0.0257 | 0.0083 |
| Heart V20 (%) | 0.0192 | 0.0096 |
| N Stage | 0.0139 | 0.0057 |
| Heart V50 (%) | 0.0125 | 0.0024 |
| M Stage | 0.0106 | 0.0065 |
| Tumor_Histology | 0.0102 | 0.0032 |
| Heart V40 (%) | 0.0098 | 0.0047 |
| Site of Primary | 0.0096 | 0.0034 |
| T Stage | 0.009 | 0.0034 |
| Heart V30 (%) | 0.0085 | 0.0062 |
| Stage at Dx | 0.0062 | 0.0029 |
| RT1 Dose (in Gy) | 0.0053 | 0.0036 |
| Smoking | 0.0052 | 0.0018 |
| Sex | 0.0049 | 0.0023 |
| Immunotherapy? | 0.0048 | 0.0019 |
| RT1 Fractions | 0.0014 | 0.001 |
| Race_Ethnicity | 0 | 0 |
| Surgery? | -0.001 | 0.0016 |

| **Model** | **AUC, 95% CI** | **Sensitivity, 95% CI** | **Specificity, 95% CI** | **F1, 95% CI** |
| --- | --- | --- | --- | --- |
| Logistic Regression (LR) | 0.714, [0.59, 0.828] | 0.712, [0.585, 0.833] | 0.647, [0.481, 0.796] | 0.733, [0.621, 0.819] |
| Random Forest Model (RF) | 0.696, [0.574, 0.811] | 0.827, [0.723, 0.923] | 0.559, [0.393, 0.724] | 0.782, [0.686, 0.86] |
| Gradient Boosting Machine (GBM) | 0.619, [0.486, 0.742] | 0.750, [0.629, 0.860] | 0.559, [0.389, 0.724] | 0.736, [0.63, 0.824] |
| Support Vector Machine (SVM) | 0.682, [0.565, 0.794] | 0.731, [0.605, 0.851] | 0.618, [0.457, 0.774] | 0.738, [0.636, 0.826] |

**ML Supplementary Table S6:** Discrimination metrics and the performance of the reduced features ML models predicting mortality after CRT in Appalachian lung cancer population.

(a)

(b)

**ML Supplementary Figure S3:** Performance and feature importance of Logistic Regression Features-reduced model predicting mortality in patients with lung cancer received chemoradiation. **(a)** Area Under the Receiver Operating Curve (AUC) of four models. **(b)** Feature importance of the Logistic Regression Model predicting mortality during and after definitive CRT in lung cancer population using permutation feature importance.

**ML Supplementary Table S7:** Feature importance of Logistic Regression reduced features model predicting mortality in lung cancer during or after chemoradiation.

| Variable | Mean AUC Decrease | SD |
| --- | --- | --- |
| Mean Heart (Gy) | 0.1144 | 0.0352 |
| Heart V20 (%) | 0.0868 | 0.0402 |
| M Stage | 0.0678 | 0.0309 |
| Heart V40 (%) | 0.0585 | 0.0323 |
| Heart V50 (%) | 0.0572 | 0.0209 |
| Age_at_Diagnosis | 0.0569 | 0.0291 |
| Cardiovascular comorbidity | 0.0545 | 0.0252 |
| N Stage | 0.002 | 0.0049 |
| Smoking | -0.0007 | 0.003 |
| Tumor_Histology | -0.0008 | 0.0036 |
| Heart V30 (%) | -0.0046 | 0.0119 |
